# Deciphering trait associated morpho-physiological responses in pearlmillet hybrids and inbred lines under salt stress

**DOI:** 10.3389/fpls.2023.1121805

**Published:** 2023-03-02

**Authors:** Ashwani Kumar, Parvender Sheoran, Anita Mann, Devvart Yadav, Arvind Kumar, Sunita Devi, Naresh Kumar, Pooja Dhansu, Dinesh K. Sharma

**Affiliations:** ^1^ Division of Crop Improvement, ICAR-Central Soil Salinity Research Institute, Karnal, India; ^2^ Division of Social Sciences Research, ICAR-Central Soil Salinity Research Institute, Karnal, India; ^3^ Chaudhary Charan Singh Haryana Agricultural University, Hisar, India; ^4^ Department of Chemistry and Biochemistry Eternal University, Baru, Sahib, India; ^5^ ICAR–Sugarcane Breeding Institute, Regional Center, Karnal, India

**Keywords:** saline irrigation, salt tolerance, morpho–physiological traits, regression analysis, trait modeling, pearl millet yield

## Abstract

Pearl millet is a staple food for more than 90 million people residing in highly vulnerable hot arid and semi–arid regions of Africa and Asia. These regions are more prone to detrimental effects of soil salinity on crop performance in terms of reduced biomass and crop yields. We investigated the physiological mechanisms of salt tolerance to irrigation induced salinity stress (EC_iw_ ~3, 6 & 9 dSm^–1^) and their confounding effects on plant growth and yield in pearl millet inbred lines and hybrids. On average, nearly 30% reduction in above ground plant biomass was observed at EC_iw_ ~6 dSm^-1^ which stretched to 56% at EC_iw_ ~9 dSm^-1^ in comparison to best available water. With increasing salinity stress, the crop performance of test hybrids was better in comparison to inbred lines; exhibiting relatively higher stomatal conductance (gS; 16%), accumulated lower proline (Pro; –12%) and shoot Na^+^/K^+^(–31%), synthesized more protein (SP; 2%) and sugars (TSS; 32%) compensating in lower biomass (AGB; –22%) and grain yield (GY: –14%) reductions at highest salinity stress of EC_iw_ ~9 dSm^–1^. Physiological traits modeling underpinning plant salt tolerance and adaptation mechanism illustrated the key role of 7 traits (AGB, Pro, SS, gS, SPAD, Pn, and SP) in hybrids and 8 traits (AGB, Pro, PH, Na^+^, K^+^, Na^+^/K^+^, SPAD, and gS) in inbred lines towards anticipated grain yield variations in salinity stressed pearl millet. Most importantly, the AGB alone, explained >91% of yield variation among evaluated hybrids and inbreed lines at EC_iw_ ~9 dSm^–1^. Cumulatively, the better morpho–physiological adaptation and lesser yield reduction with increasing salinity stress in pearl millet hybrids (HHB 146, HHB 272, and HHB 234) and inbred lines (H77/833–2–202, ICMA 94555 and ICMA 843–22) substantially complemented in increased plant salt tolerance and yield stability over a broad range of salinity stress. The information generated herein will help address in deciphering the trait associated physiological alterations to irrigation induced salt stress, and developing potential hybrids in pearl millet using these parents with special characteristics.

## 1 Introduction

Pearl millet [*Pennisetum glaucum* (L.) R. Br.] is a C4 type, small–grained cereal crop occupying ~26 million ha (m ha) area worldwide, provides food and nutritional security to millions of people inhabiting arid and semi–arid regions (Yadav et al., 2012; Shivhare and Lata, 2017). In India, pearl millet is the fourth most extensively cultivated food crop after rice, wheat and maize with 6.93 m ha area and 1.2 t ha^–1^ average productivity (Directorate of Millets Development, 2020). Being hardy and robust in nature, pearl millet could survive under multiple abiotic stresses particularly drought, heat and alkalinity/salinity (Yadav et al., 2012a; Toderich et al., 2018). Given its high nutritional (Zn and Fe) value, well balanced amino acid profile and rich source of insoluble dietary fiber, pearl millet is better suited for drier areas fulfilling both food and feed requirements. These adaptive and nutritional features make pearl millet an important crop that can effectively address the emerging and intersecting challenges of global warming, water crisis, land degradation and food-related health issues. Despite these innate benefits and exceptional buffering capacity against harsh climatic conditions, the resilience and sustainability issues in pearl millet production still demand for development of new plant types with multiple stress tolerance, efficient resource use and yield stability amidst unprecedented effects of climate change and associated environmental hazards.

Dryland salinity is one of the major abiotic constraints negatively affecting the plant growth and crop productivity. Moreover, the natural and anthropological factors and increased dependency on marginal quality underground water for irrigation further accelerates the process of soil salinization. In consequence, nearly 10 million hectares of land gets salinized every year at global scale, and if the current trend continues unabated, ~16.2 million hectares area in India only will be degraded by different degrees of soil salinization by 2050 (Sharma et al., 2015). Salinity induced changes in morpho–physiological processes cause several inhibitory effects on plant growth and development by dint of restricted nutrient uptake, partial stomata closure, unbalanced ion homeostasis and cell membrane injury, and ultimately reduced crop yields (Wang et al., 2014; Dhansu et al., 2021; Kumar et al., 2021). Identifying specific variability in these morpho-physiological characteristics associated with plant salt tolerance is highly complex; being polygenic in nature and influenced by genetic and environmental factors. Therefore, interlinking physiological basis of salt tolerance might help improve our understanding in underpinning the plant tolerance mechanisms and stabilizing crop productivity in degraded environments.

Under these circumstances, the management options including improved irrigation techniques offer tremendous potential to prevent and mitigate the adverse effects of soil salinization, but often remain prohibitively expensive. Contrarily, varietal adaptation strategy seems to be less expensive and more viable in bridging the yield gaps and harnessing the agricultural potential in stress–prone areas (Sheoran et al., 2021; Soni et al., 2021). Several studies on genotypic variability for salinity tolerance have been documented in cereal crops including pearl millet (Maiti and Satya, 2014; Shivhare and Lata, 2017). However, a better understanding of the plant physiological mechanisms governing the plant salt tolerance, prioritization of important traits of interest, and identification of superior parent lines/hybrids are of great interest in breeding programs related to marginal environments. With this hypothesis, the present investigation was carried out to define the relevance of physiological trait associated variability and their contribution in plant salt tolerance, and identify the best performing inbred lines/hybrids to stabilize pearl millet production over a broad range of irrigation induced salinity stress.

## 2 Material and methods

### 2.1 Experimental setup and treatment details

Pearl millet genotypes consisting of 10 inbred lines and 7 hybrids (**Supplementary Table 1**) were collected from CCS Haryana Agricultural University, Hisar, Haryana. These genotypes were evaluated under controlled conditions in factorial randomized complete block design with five replications at ICAR–Central Soil Salinity Research Institute, Karnal (29°43’ N latitude, 76°58’ E longitude) during kharif season of 2018-19. The seeds were sown in 20 kg capacity porcelain pots, filled with 16 kg saline soil (EC_e_ ~ 5.98 dSm^–1^, bulk density: 1.45 g cc^–1^ porosity: ~40%). Nine seeds per pot were sown at 3–4 cm depth and these pots were immediately saturated with deionized water upto the field capacity (28% v/v). After 15 days of seedlings emergence, thinning was done to retain three seedlings per pot for imposing the salt stress treatments and recording biometric observations. Thereafter, the osmotic and ionic stress was imposed by applying three levels of saline irrigations as mild salinity of EC_iw_ ~3 dSm^–1^, moderate salinity of EC 6 dSm^–1^ and high salinity of EC 9 dSm^–1^ with one additional set of plants as control irrigated with best available water (BAW) having EC_iw_ ~0.6 dSm^–1^. Treatment–wise need based saline irrigations were applied as per crop requirement up to physiological maturity. The soil salinity was maintained through saline irrigations of EC_iw_ 3, 6, and 9 dSm^-1^ during the plant growing season. The salinity build-up during the experiment was monitored by analysis of soil EC at initial (before sowing and saline irrigation) and final (at harvesting of the crop) stages (**Supplementary Figure 1**). The pot house was covered with high density transparent polythene sheet to prevent the entry of rain water, and also to maintain the desired levels of salinity stress.

### 2.2 Physiological and biochemical observations and estimations

Salinity–induced changes in physiological parameters of crop growth were recorded at anthesis stage. The changes in quantitative variables; plant height (PH, cm), above ground biomass (AGB, g plant^–1^), photosynthetic rate (Pn, µmol m^–2^ s^–1^), stomatal conductance (gS, mol m^–2^ s^–1^), transpiration rate (E, mmol m^–2^ s^–1^), membrane injury (MI, %), total chlorophyll content (CC, mg g^–1^), soil plant analysis development (SPAD) chlorophyll meter reading measuring the greenness of the leaves, relative water content (RWC, %), proline (Pro, mg g^–1^), total soluble sugars (TSS, mg g^–1^), total soluble protein (TSP, g g^–1^), sodium content (Na^+^, %), potassium content (K^+^, %), sodium to potassium (Na^+^/K^+^) ratio were recorded accordingly to understand their influences on grain yield (GY, g plant^–1^) under variable salinity stress.

#### 2.2.1 Gas exchange parameters

The observations on gas exchange parameters; photosynthetic rate (Pn), stomatal conductance (gS) and transpiration rate (E) were recorded on five randomly tagged, fully expanded flag leaves through portable gas exchange system Infra-red gas analyzer (LICOR–6400XT, LICOR Inc., Lincoln, NE). Measurements were performed at 25°C cuvette temperature, 1100 µmol m^–2^ s^–1^ PAR, and ambient CO_2_ of 400 ppm growth environments, respectively.

#### 2.2.2 Total chlorophyll content

Total chlorophyll content was determined by incubating 100 mg flag leaf tissue in 10 ml DSMO solvent for 1 hr at 60–65°C in a water bath. After tissue decolorization, the solvent was cooled down at room temperature (for 30 min), and absorbance was measured at 665 nm and 648 nm with a UV–VIS spectrophotometer (Electronics, India). Total chlorophyll concentration was expressed as mg g^-1^ fresh weight and was estimated according to Barnes et al. (1992).


$$\text{Total chlorophyll content}\left({\text{mg g}}^{-1}\right)=\left(7.49*A_{665}+20.34*A_{648}\right)\times \left[V/\left(1000*\text{W}\right)\right]$$

Where, A_665_ is the absorbance measured at 665 nm wavelength, and A_648_ is the absorbance measured at 648 nm wavelength; V is the final volume of the solvent (ml); and W is the weight of leaf tissue (mg).

#### 2.2.3 SPAD index

SPAD 502 PLUS (KONICA MINOLTA) was used to measure the SPAD readings in both control and salt treated plants. It was measured on the same leaf which was taken for chlorophyll estimation.

#### 2.2.4 Relative water content

The representative leaf samples were collected and immediately weighed to record the fresh weight (FW). These leaves were then immersed in distilled water for 4 hrs in closed petri dishes for estimating the turgid weight (TW), and kept thereafter for drying in pre–heated hot air oven at 60°C for 72 hrs or till attainment of constant dry weight (DW). The RWC was calculated according to the formula given by Weatherley (1950).


RWC (%)=[(FW−DW)/(TW−DW)]∗100

#### 2.2.5 Membrane injury

Membrane injury was determined by following the method of Dionisio-Sese and Tobita (1998). The sample leaves were washed properly in distilled water and individually cut into small pieces of 1 cm size, and then immersed in 10 ml deionised water at 25°C. After 5 hrs, the electrical conductivity (EC) of the solution was measured using the EC meter (CON 700, Eutech, India) and designated as EC_a_. Total rupturing of plant tissue was attained by keeping the sampled leaves in boiling water bath (100°C) for 60 min. After cooling, the EC of the solution was again measured and designated as EC_b_. The MI was calculated using the following formula and expressed in per cent:


$$\text{MI}\left(\%\right)=\left[{\text{EC}}_{\text{a}}/\left({\text{EC}}_{\text{a}}{+\text{EC}}_{\text{b}}\right)\right]*100$$

#### 2.2.6 Osmolytes accumulation

Fresh leaves were collected between 9–10 AM and immediately sealed in humified polythene bags, weighed and analyzed for osmolytes accumulation following the standard analytical procedures for estimation of proline (Bates et al., 1973), total soluble protein (Bradford, 1976) and total soluble sugars (Yemm and Willis, 1954).

##### 2.2.6.1 Proline

Fresh leaves (200 mg) were homogenized in 5 ml solution of 3% sulphosalicylic acid. After centrifugation at 10,000 rpm for 10 min at 4°C, 2 ml of supernatant was added to a test tube containing 2 ml of acid ninhydrin reagent (1.25 g ninhydrin dissolved in 20 ml of 6*N* O–phosphoric acid and 30 ml of glacial acetic acid) and 2 ml of glacial acetic acid and incubated at 100°C for 1 hr. After cooling, 4 ml of toluene was added and vortexed. Absorbance was measured at 520 nm wavelength using upper phase on UV spectrophotometer (SPECORD 210 PLUS) with toluene as blank. Proline content was calculated by using the standard curve of different concentrations of L–proline.

##### 2.2.6.2 Total soluble protein

Fresh leaves (1 g) were crushed in 2.5 ml of chilled tris buffer (pH 8.0, 0.1 M) having 0.1% PVP (polyvinyl pyrrolidone) to prepare a sample extract. A reaction mixture was prepared by adding 2.5 ml Bradford reagent (ready to use) and 50 µl of protein sample. Absorbance of the protein mixture was measured at 595 nm wavelength after 10 min and the protein content was determined using a standard curve of Bovine Serum Albumin (BSA).

##### 2.2.6.3 Total soluble sugars

Fresh leaves (100 mg) were crushed in 2.5 ml of 80% ethanol, and centrifuged at 10,000 rpm for 10 min at 4°C. Supernatant (100 µl) was pipetted in a test tube containing 5 ml of anthrone reagent (0.4% anthrone prepared in chilled concentrated sulphuric acid) and incubated at 100°C for 10 min. Thereafter, the absorbance was measured at 620 nm wavelength on UV spectrophotometer using anthrone reagent as blank. Standard curve of D–glucose was used for calculation of total soluble sugars as mg g^-1^ FW.

#### 2.2.7 Ionic analysis

For estimating Na^+^ and K^+^, fresh leaf samples were collected, washed with distilled water and then dried in oven at 70°C. Finely-grounded 100 mg sample was digested in 10 ml of HNO_3_:HClO_4_ (3:1 ratio) di–acid mixture, and the concentrations of Na^+^ and K^+^ were determined using NaCl and KCl as standards on flame photometer (Systronics 128, India).

### 2.3 Yield measurements and salinity buildup

The crop was harvested at physiological maturity when the leaves turned yellow and dried up, and the grains became hard and firm with a black spot in the hilar region. Treatment–wise earheads were harvested first, and the stalks were cut from the ground level, stacked and dried accordingly. The earheads were threshed manually to calculate the grain yields after adjusting it to 14% moisture content by measuring on the Seed moisture meter. To observe the changes in salinity buildup, the soil samples were collected and analyzed before sowing and just after crop harvest and measured in terms of EC_e_ (dSm^–1^).

### 2.4 Data analysis

Prior to analysis, the observed and estimated values of five plants (68 data points) under each replication of the variables were tested for their normality (Q–Q plot of residuals) through Spahiro–Wilk (W) test (Shapiro and Wilk, 1965) at the 95% confidence of interval (p>0.05) (**Supplementary Table 2**). It is used for testing the pre-requisite hypothesis (whether the random sample drawn from a normal Gaussian probability distribution (X∼N (μ,σ^2^)) for ANOVA or other parametric statistical analysis. The observations of nine variables (Plant height-PH, Relative water content-RWC, Photosynthetic rate-Pn, Membrane injury-MI, Proline content-Pro, sodium content-Na, potassium content-K, Na/K Ratio, above ground biomass-BM) were not following normal probability; therefore, log transformation was applied accordingly. Two–way analysis of variance (ANOVA) technique was employed to dissect the variability within genotype (G), salinity (S) and G × S effects for each test variable in Split Plot Design with three replications using the Generalized Linear Model with mixed effect through STAR statistical software (IRRI, 2013).


$$y_{i\;jk}=\mu +\alpha_{i}+\beta_{j}+e_{i\;j}+\;\beta_{k}+{\left(\alpha \beta \right)}_{jk}+\epsilon_{ijk}$$

Where, *i* = 3, number of blocks (replication); *j* = 4, Levels of salinity stress (0, 3, 6, and 9); *k* = 17, number of genotypes; *μ* = overall mean for particular variable; *ρ_i_* = effect of block; *α_j_* = main effect of salinity stress (fixed effect); *e_ij_* = block by salinity interaction (the whole plot error, random random); *β_k_* = main effect of genotypes (fixed); *(αβ)_jk_* = interaction between salinity and genotypes (fixed); *ϵ_ijk_* = residual effect (subplot error) (random). (**Table 1**). Multiple comparisons were performed for 84 data points in hybrids and 120 data points in inbreds using Tukey’s HSD test (Tukey, 1977) to determine the significant differences between treatments at 5% level of significance. Key morpho–physiological traits were prioritized separately among evaluated pearl millet hybrids and inbred through traits modeling using stepwise regression (backward elimination) approach in STAR statistical software. Pearson correlation coefficients (Pearson, 1948) were estimated to determine the association cumulatively across the irrigation induced salinity stress for evaluated parameters. Biplot analysis was performed manually through Microsoft excel 2016.

**Table 1:** Analysis of variance (ANOVA) for morpho–physiological traits in pearl millet under salinity stress.

Source of Variation	DF	Mean Squares							
		PH	RWC	MI	CC	SPAD	Pn	gS	E
Block	2	673.21^***^	109.66^***^	15.97^ns^	0.19^ns^	126.24^***^	0.80^ns^	0.000^ns^	0.10^*^
Salinity	3	19547.29^***^	3272.55^***^	7101.06^***^	2.55^*^	1759.32^***^	2512.01^***^	0.457^***^	132.84^***^
Error(a)	6	13.75	0.14	5.11	0.27	0.20	0.54	0.000	0.02
Genotypes	16	6076.29^***^	100.20^***^	45.18^***^	0.18^ns^	134.75^***^	70.52^***^	0.023^***^	4.62^***^
Hybrids *vs* Inbreds	1	42712.86^***^	126.29^***^	79.16^***^	0.03^ns^	37.13^***^	6.20**	0.171^***^	3.69^***^
Genotypes × Salinity	48	412.01^***^	21.24^***^	33.59^***^	0.05^ns^	26.63^***^	27.09**	0.003^***^	0.47^***^
(Hybrids *vs* Inbreds) × Salinity	3	1598.48^***^	33.79^***^	22.80^***^	0.01^ns^	96.51^***^	97.83***	0.003^***^	0.12^***^
Error(b)	128	5.95	0.15	0.22	0.12	0.16	0.35	0.000	0.02
		Pro	TSS	TSP	Na^+^	K^+^	Na^+/^K^+^	AGB	GY
Block	2	0.04 ^ns^	6.59 ^ns^	0.26 ^ns^	0.00 ^ns^	0.01 ^ns^	0.00 ^ns^	11.68^***^	0.14^ns^
Salinity	3	271.79^***^	1278.39^***^	264.25^***^	2.64^***^	5.82^***^	0.17^***^	4931.78^***^	605.03^***^
Error(a)	6	0.06	15.88	0.47	0.00	0.01	0.00	0.48	0.10
Genotypes	16	2.40^***^	85.24^***^	30.27^***^	0.60^***^	7.22^***^	0.04^***^	523.79^***^	95.07^***^
Hybrids *vs* Inbreds	1	14.28^***^	0.01^ns^	39.02^***^	0.56^***^	21.39^***^	0.03^***^	3439.36^***^	1009.08^***^
Genotypes × Salinity	48	0.14^***^	20.36^***^	9.73^***^	0.64^***^	4.33^***^	0.04^***^	71.66^***^	5.04^***^
(Hybrids *vs* Inbreds) × Salinity	3	0.32^***^	40.05^***^	11.90^***^	0.06^***^	7.62^***^	0.01^***^	147.68^***^	16.39^***^
Error(b)	128	0.02	0.38	0.19	0.00	0.02	0.00	0.10	0.05

***significant at p<0.001; **significant at p<0.01; *significant at p<0.05; ns, non–significant; DF, degree of freedom; PH, plant height (cm); RWC, relative water content (%); MI, membrane injury (%); CC: chlorophyll content (mg g^–1^), SPAD, soil plant analysis development (SPAD) chlorophyll meter reading; Pn, photosynthetic rate (µmol m^-2^ s^-1^); gS, stomatal conductance (mol m^-2^ s^-1^); E, transpiration rate (mmol m^-2^ s^-1^); Pro, proline content (mg g^–1^); TSP: total soluble protein (mg g^–1^); SS, total soluble sugars (mg g^–1^); Na^+^, sodium content (%); K^+^, potassium content (%); Na^+^/K^+^, sodium to potassium ratio; AGB, above ground biomass (g plant^–1^); GY, grain yield (g plant^–1^).

## 3 Results

Exposure to irrigation induced salinity stress was evaluated in terms of morphological (growth and yield) and physiological (plant water status, gas exchange, osmolytes accumulation and ionic balance) parameters of crop growth in pearl millet. Our results indicated that the effects of saline irrigation water, tested genotypes (hybrids and inbred lines) and their interactions were highly significant (p<0.01 & 0.05) for all the evaluated parameters (**Table 1**).

The group comparison (hybrids *vs* inbred lines) analysis (**Table 2**) revealed test hybrids performed better while maintaining higher stomatal conductance (gS; 16% and transpiration rate (E; 5%), lower proline accumulation (Pro; –10%) and ionic balance (Na^+^/K^+^ ratio; –20%) in shoot portion under prevalent salinity stress culminating in better crop performance (ABG; 368% and GY; 82%). With increasing salinity stress, significant reduction in expression of all the morphological traits was noticed where we can see more than 40% decrease in grain yield (48%) biomass (40%) in inbreds and less than 30% change in physiological traits like chlorophyll content, SPAD index, photosynthetic rate etc. Similarly, most of the physiological parameters decreased significantly, except for MI, Pro, TSP, Na^+^ and Na^+^/K^+^, which significantly (p<0.05) increased in response to increasing salinity stress levels (**Table 3**).

**Table 2:** Effects of salinity stress on various traits in pearl millet hybrids and inbreds through group comparison analysis (averaged across 7 hybrids and 10 inbred lines).

Traits	Units	Hybrids		Inbreds		F_cal_	*p*>(F)
		Mean	% change over control	Mean	% change over control		
Plant height (PH)	cm	128.8 ± 12.9	-11.04	96.7 ± 30.8	-25.82	7178.4	0.0000
Relative water content (RWC)	%	82.9 ± 8.4	-12.44	84.1 ± 7.5	-10.84	838.9	0.0000
Membrane injury (MI)	%	19.5 ± 11.2	-27.26	18.40 ± 10.6	-25.88	355.6	0.0000
Chlorophyll content (CC)	mg g^–1^ FW	1.09± 0.39	-20.49	1.08± 0.38	-14.05	0.30	0.5874
SPAD	–	46.5 ± 7.5	-30.11	45.5 ± 5.9	-35.38	236.4	0.0000
Photosynthetic rate (Pn)	µmol CO_2_ m^–2^ s^–1^	20.8 ± 5.9	-23.48	21.7 ± 7.7	-31.66	17.7	0.0000
Stomatal conductance (gS)	mol H_2_O m^–2^ s^–1^	0.43± 0.08	-28.85	0.37± 0.10	-31.08	1835.6	0.0000
Transpiration rate (E)	mmol H_2_O m^–2^ s^–1^	6.18± 1.50	196.59	5.90± 1.61	150.77	152.4	0.0034
Proline content (Pro)	mg g^–1^ FW	5.19	3.86± 2.08	177.96	681.0	0.0000
Total soluble sugars (TSS)	mg g^–1^ FW	17.8 ± 4.2	111.75	17.4 ± 6.4	124.76	0.03	0.8695
Total soluble protein (TSP)	mg g^–1^ FW	10.3 ± 2.8	9.94	10.9 ± 3.0	-15.07	203.8	0.0000
Sodium content (Na^+^)	%	0.36± 0.51	96.12	0.50± 0.47	201.37	1307.2	0.0000
Potassium content (K^+^)	%	4.76± 0.74	56.18	5.52± 1.51	36.85	893.8	0.0000
Sodium to potassium ratio (Na^+^/K^+^)	–	0.08± 0.11	-28.19	0.10± 0.13	-34.41	1442.4	0.0000
Above ground biomass (AGB)	g plant^–1^	36.8 ± 8.4	-24.36	27.1 ± 11.7	-40.5	33610.9	0.0000
Grain yield (GY)	g plant^–1^	10.9 ± 3.8	-35.7	6.0± 3.1	-48.75	18444.5	0.0000

Soil Plant Analysis Development (SPAD) chlorophyll meter reading; Data represents mean value of 30 pooled measurements (4 levells of salinity × 3 plants per pot × 5 replications); ± indciate standard deviation from the mean value.Negative sign in percent cahnge values shows decrease w.r.t control.

**Table 3:** Effect of salinity stress on morpho–physiological attributes of crop growth and yield in pearl millet (averaged across 7 hybrids and 10 inbred lines).

Irrigation water salinity	Traits							
	PH	RWC	MI	CC	SPAD	Pn	gS	E
BAW	128.35^a^	91.48^a^	8.32^d^	1.35^a^	52.54^a^	28.46^a^	0.50^a^	7.77^a^
EC_iw_ ~3 dSm^–1^	121.31^a^	87.68^b^	11.35^c^	1.09^b^	48.41^b^	25.67^b^	0.44^b^	6.72^b^
EC_iw_ ~6 dSm^–1^	105.74^b^	82.16^c^	21.18^b^	1.09^b^	43.78^c^	18.17^c^	0.36^c^	5.55^c^
EC_iw_ ~9 dSm^–1^	84.18^c^	73.03^d^	34.51^a^	0.8^c^	38.93^d^	13.08^d^	0.28^d^	4.01^d^
	Pro	TSS	TSP	Na^+^	K^+^	Na^+/^K^+^	AGB	GY
BAW	1.49^d^	8.03^d^	23.07^a^	0.23^c^	5.48^a^	0.04^b^	41.39^a^	11.67^a^
EC_iw_ ~3 dSm^–1^	2.55^c^	9.88^c^	18.71^b^	0.30^bc^	5.17^ab^	0.06^b^	35.49^b^	9.54^b^
EC_iw_ ~6 dSm^–1^	3.94^b^	13.39^a^	17.45^b^	0.49^b^	5.42^a^	0.10^b^	29.00^c^	7.16^c^
EC_iw_ ~9 dSm^–1^	6.82^a^	11.43^b^	10.96^c^	0.74^a^	4.74^b^	0.17^a^	18.43^d^	3.63^d^

Means followed by similar lowercase letter within a column for a partiuclar trait are not statistically significant (p<0.05) using Tukey’s HSD test; Data represents mean value of 255 pooled measurements (17 genotypes × 3 plants per pot × 5 replications); BAW, best available water; EC_iw_, electrical conductivity of irrigation water; dSm^–1^, deci siemens per meter; PH, plant height (cm); RWC, relative water content (%); MI, membrane injury (%); CC, chlorophyll content (mg g^–1^), SPAD, soil plant analysis development (SPAD) chlorophyll meter reading; Pn, photosynthetic rate (µmol m^-2^ s^-1^); gS, stomatal conductance (mol m^-2^ s^-1^); E, transpiration rate (mmol m^-2^ s^-1^); Pro, proline content (mg g^–1^); TSP, total soluble protein (mg g^–1^); TSS, total soluble sugars (mg g^–1^); Na^+^, sodium content (%); K^+^, potassium content (%); Na^+^/K^+^, sodium to potassium ratio; AGB, above ground biomass (g plant^–1^); GY, grain yield (g plant^–1^).

### 3.1 Plant height, leaf water status and chlorophyll content

Progressive decline in plant height (PH) was noticed with each gradual increase in salinity stress; albeit to a greater extent in inbred lines compared to the hybrids. On an average, the PH reduced by 6%, 18% and 34% with irrigation water salinity (EC_iw_) of 3, 6 and 9 dSm^–1^, respectively (**Table 3**). Across salinity levels, hybrid HHB 146 attained maximum PH (137 cm) (**Supplementary Figure 2**) while minimum was observed in HHB 226 (124 cm). Within inbred lines, the PH ranged from 64 cm (HMS 7A) to 124 cm (ICMA 97111) (**Table 4**).

**Table 4:** Mean response of evaluated hybrids and inbred lines for plant water relations, gas exchange, osmolyte accumulation, ionic balance and yield parametes in pearl millet (averaged across salinity levels).

Traits	PH	RWC	MI	CC	SPAD	Pn	gS	E	Pro	TSP	TSS	Na^+^	K^+^	Na^+^/K^+^	AGB	GY
Hybrids																
HHB 67 Improved	128.0^cd^	78.9^c^	20.4^b^	1.06^c^	44.3^d^	23.6^a^	0.46^a^	6.51^a^	2.85^e^	8.31^d^	17.19^d^	0.54^b^	5.01^a^	0.106	32.8^e^	10.0^e^
HHB 146	137.3^a^	85.7^a^	17.3^d^	0.93^a^	43.9^d^	22.2^b^	0.45^a^	6.59^a^	3.93^a^	9.81^c^	19.25^ab^	0.16^e^	5.02^a^	0.032^f^	40.3^b^	12.4^b^
HHB 197	129.5^bc^	79.5^c^	17.7^cd^	1.05^a^	48.2^b^	21.2^c^	0.46^a^	6.23^b^	3.52^c^	11.20^a^	18.39^bc^	0.86^a^	4.44^c^	0.190^a^	32.4^e^	9.1^f^
HHB 226	124.1^d^	85.9^a^	20.5^b^	1.32^a^	46.9^c^	22.4^b^	0.44^b^	6.51^a^	3.38^d^	10.31^bc^	19.99^a^	0.23^d^	4.27^d^	0.056^d^	34.1^d^	9.4^f^
HHB 223	124.4^d^	82.3^b^	18.3^c^	0.85^a^	43.3^e^	18.1^d^	0.38^d^	5.92^c^	3.76^b^	10.81^ab^	18.24^c^	0.23^d^	5.08^a^	0.040^e^	37.4^c^	10.7^d^
HHB 234	133.0^ab^	82.2^b^	21.5^a^	0.93^a^	47.4^c^	16.9^e^	0.41^c^	5.86^c^	3.93^a^	10.69^ab^	15.75^e^	0.36^c^	4.78^b^	0.074^c^	43.2^a^	12.9^a^
HHB 272	125.1^cd^	85.7^a^	20.5^b^	1.15^a^	50.7^a^	21.0^c^	0.41^c^	5.64^d^	2.88^e^	11.05^a^	15.50^e^	0.18^e^	4.74^b^	0.038^ef^	37.3^c^	11.9^c^
Inbred lines																
ICMA 97111	123.9^a^	82.9^e^	15.6^e^	1.07^a^	51.9^a^	24.1^ab^	0.42^a^	5.99^c^	4.02^bcd^	8.84^d^	15.24^e^	0.64^c^	4.72^f^	0.134^c^	29.1^c^	6.5^c^
ICMA 843-22	84.0^f^	81.2^g^	19.6^c^	0.96^a^	46.2^e^	22.5^cd^	0.38^cd^	6.78^a^	4.05^abc^	10.83^c^	15.87^de^	0.43^f^	5.01^ef^	0.081^f^	32.0^b^	6.0^d^
ICMA 94555	76.4^g^	81.9^f^	17.6^d^	1.00^a^	43.8^g^	25.1^a^	0.40^b^	6.95^a^	3.85^d^	8.43^d^	16.70^d^	0.32^h^	6.02^b^	0.048^h^	28.5^d^	6.1^d^
HMS 7A	64.3^h^	80.8^g^	18.0^d^	0.95^a^	46.8^d^	22.3^cd^	0.36^ef^	5.79^c^	4.16^ab^	14.16^a^	18.18^c^	0.39^g^	5.30^de^	0.087^ef^	26.6^f^	6.0^d^
HMS 47A	90.8^e^	90.0^a^	22.2^a^	1.06^a^	41.4^h^	18.3^f^	0.34^f^	4.90^e^	4.23^a^	12.19^b^	24.02^a^	0.79^a^	5.36^cd^	0.216^a^	18.3^i^	4.1^f^
ICMA 95222	119.8^b^	82.9^e^	18.0^d^	1.01^a^	41.3^h^	22.7^cd^	0.39^bc^	6.04^c^	3.18^e^	12.16^b^	15.47^e^	0.76^b^	5.66^c^	0.169^b^	34.8^a^	7.9^a^
HBL 11	80.1^g^	87.3^b^	18.0^d^	1.14^a^	49.5^b^	17.4^f^	0.30^g^	5.27^d^	4.1^abc^	11.07^c^	21.46^b^	0.20^i^	5.03^ef^	0.039^i^	21.6^h^	4.7^e^
H77/833-2-202	106.7^d^	83.7^d^	19.4^c^	1.14^a^	45.1^f^	22.0^d^	0.37^de^	6.02^c^	3.94^cd^	12.46^b^	18.36^c^	0.46^e^	5.52^cd^	0.089^e^	29.0^c^	7.3^b^
AC 04-13	106.3^d^	85.2^c^	20.3^b^	1.03^a^	48.4^c^	23.2^bc^	0.40^b^	6.49^b^	3.92^cd^	10.83^c^	12.89^f^	0.56^d^	7.70^a^	0.071^g^	27.8^e^	6.5^c^
HTP 94/54	114.4^c^	84.9	15.3^e^	1.03^a^	40.4^i^	19.9^e^	0.35^f^	4.76^e^	3.16^e^	8.43^d^	15.84^de^	0.42^f^	4.83^f^	0.116^d^	23.3^g^	4.7^e^

Means followed by at least one letter common (for hybrids and inbred lines) are not statistically significant (p<0.05) using Tukey’s HSD test; PH, plant height (cm); RWC, relative water content (%); MI, membrane injury (%); CC, chlorophyll content (mg g^–1^), Pn, photosynthetic rate (µmol m^-2^ s^-1^); gS, stomatal conductance (mol m^-2^ s^-1^); E, transpiration rate (mmol m^-2^ s^-1^); Pro, proline content (mg g^–1^); TSP, total soluble protein (mg g^–1^); TSS, total soluble sugars (mg g^–1^); Na^+^, sodium content (%); K^+^, potassium content (%); Na^+^/K^+^, sodium to potassium ratio; AGB, above ground biomass (g plant^–1^); GY, grain yield (g plant^–1^).

Increasing salt concentration reduced the leaf relative water content (RWC); being 91.5% in control treatment which gets decreased to 73.0% at EC_iw_ ~9 dSm^–1^ (**Table 3**). Genotypic variations and their differential response to irrigation induced salinity stress also revealed significant differences in leaf RWC; remained highest in inbred lines HMS 7A (90%) and HBL–11 (87%) compared to hybrids HHB 272, HHB 226 and HHB 146 (85%) (**Table 4**).

In the present study, salinity stress caused membrane injury (MI) to the extent of 11%, 21% and 35% with saline irrigation of EC_iw_ ~3, 6 and 9 dSm^–1^, respectively in comparison to control (**Table 3**). In general, the pearl millet inbred lines were less prone to oxidative damage (MI) with increasing salt stress than hybrids. Wide variations in MI was noticed due to irrigation induced salinity stress (**Table 4**); where, only 6 inbred lines (ICMA 97111, ICMA 94555, HMS 7A, ICMA 95222, HBL11, HTP 94154) and 3 hybrids (HHB 146, HHB 197, HHB 223) had MI <19% (the mean value of MI across salinity stresses). Lowest MI was observed in HTP 94/54 (15%) while the highest in HMS 47A (22%).

Chlorophyll content (CC), an important trait affecting the photosynthetic capacity of plant tissues, showed significant reductions of 16%, 24% and 39% when irrigated with saline water (EC_iw_) of 3, 6 and 9 dSm^–1^, respectively in comparison to control (**Table 3**). The highest CC was observed in HHB 226 (1.32 mg g^–1^) followed by HHB 272 (1.15 mg g^–1^) and HBL 11, H77/833–2–2 (1.14 mg g^–1^), while the total chlorophyll content ranging between 0.85–0.95 mg g^–1^ was observed in others, HHB 223, HHB 234, HHB 146, HMS 7A and ICMA 843–2 (**Table 4**). Highest SPAD value was observed in inbred line ICMA 97111 followed by hybrid line HHB 272 (**Table 4**). Similar to chlorophyll content, SPAD values also decreased with increasing levels of salinity with significant changes in range of 8.5 -12.5% (**Table 3**). In hybrids, the SPAD values decreased by 30% whereas in inbred lines 35% reduction was observed (**Table 2**).

### 3.2 Gas exchange parameters

Plants exposure to salinity stress exhibited significant changes in gas exchange parameters elucidating 54% reduction in photosynthetic rate (Pn), 44% reduction in stomatal conductance (gS) and 48% reduction in transpiration rate (E) at EC_iw_ ~9 dSm^–1^ (**Table 3**). Differential genotypic responses to gas exchange parameters were noticed among evaluated pearl millet genotypes. Notably, only four hybrids (HHB 226, HHB 197, HHB 146, HHB 67 Improved) and two inbred lines (AC 04–13 and ICMA 94555) had cumulative response more than their average values of Pn >21.4 µmol m^–2^ s^–1^, gS >0.40 mol m^–2^ s^–1^ and E >6.01 mmol m^–2^ s^–1^. The highest Pn of >24 µmol m^–2^ s^–1^ was observed in inbred lines ICMA 97111 and ICMA 94555 (**Table 4**). Due apparently, the evaluated hybrids had lower photosynthetic rate than the inbred lines.

### 3.3 Osmolyte accumulation

Increasing salinity stress triggered the accumulation of proline (Pro), total soluble protein (TSP) and total soluble sugars (TSS). On an average, 1.7, 2.6 and 4.6–fold increase in Pro accumulation was observed at EC_iw_ ~3, 6 and 9 dSm^–1^, respectively as against the mean value of 1.5 mg g^–1^ in control treatment (**Table 3**). By comparison, pearl millet inbred lines showed higher Pro accumulation (3.2–4.2 mg g^–1^) than the hybrids (2.9–3.9 mg g^–1^). All the inbred lines except ICMA 95222 and 3 hybrids (HHB 223, HHB 234 and HHB 146) had Pro content >3.7 mg g^–1^ (mean value across salinity stress). The maximum Pro of 4.32 mg g^-1^ was observed in inbred line HMS 47A. Similarly, maximum TSP was observed in HMS 7A (14.2 mg g^–1^) followed by H77/833–2–202, HMS 47A and ICMA 95222 (>12 mg g^–1^). Plants accumulated higher TSP with increasing salinity stress; being 23% higher at EC_iw_ ~3 dSm^–1^ and 67% higher at EC_iw_ ~6 dSm^–1^. However, further increase in salinity stress (EC_iw_ ~9 dSm^–1^) caused reduction in TSP compared to preceeding level but it was relatively higher (42%) than the control. Under stress conditions, the TSS decreased by 59%, 71% and 159% at EC_iw_ of 3, 6 and 9 dSm^–1^, respectively (**Table 4**).

### 3.4 Ionic balance

Salinity stress increased shoot Na^+^ concentration to the extent of 0.30%, 0.49% and 0.73% with saline water irrigations of EC_iw_ ~3, 6 and 9 dSm^–1^. No significant changes were observed for K^+^ content upto EC_iw_ ~6 dSm^–1^; however, further increase in salinity stress (EC_iw_ ~9 dSm^–1^) reduced K^+^ content by 13.5% (**Table 3**). Wide variations in shoot Na^+^ was observed in evaluated genotypes; notably only one inbred line (HBL 11) and 4 hybrids (HHB 146, HHB 223, HHB 226 and HHB 272) had Na^+^ accumulation <0.23%. It is interesting to note that inbred lines showed more K^+^ affinity than the hybrids. Within inbred lines, highest K^+^ accumulation was observed in AC 04–13 (7.7%) and lowest in ICMA 97111 (4.7%). All inbred lines except HTP 94/54 and ICMA 843–22 had shoot K^+^ >5.2%, the average value across variable salinity stress. Among hybrids, HHB 223 had the highest K^+^ accumulation (5.1%) while lowest was recorded in HHB 226 (4.3%). Evidently, none of the hybrid showed K^+^ accumulation more than 5.2% (**Table 4**). Out of 17 genotypes, two hybrids (HHB 67 Improved and HHB 234) and three inbred lines (HMS 7A, HMS 47A and ICMA 95222) showed increasing Na^+^/K^+^ trend with increasing stress intensity, while rest exhibited a gradual increase only upto EC_iw_ 6 ~dSm^–1^ and a declining trend was observed thereafter.

### 3.5 Yield assessment

**Figure 1:**
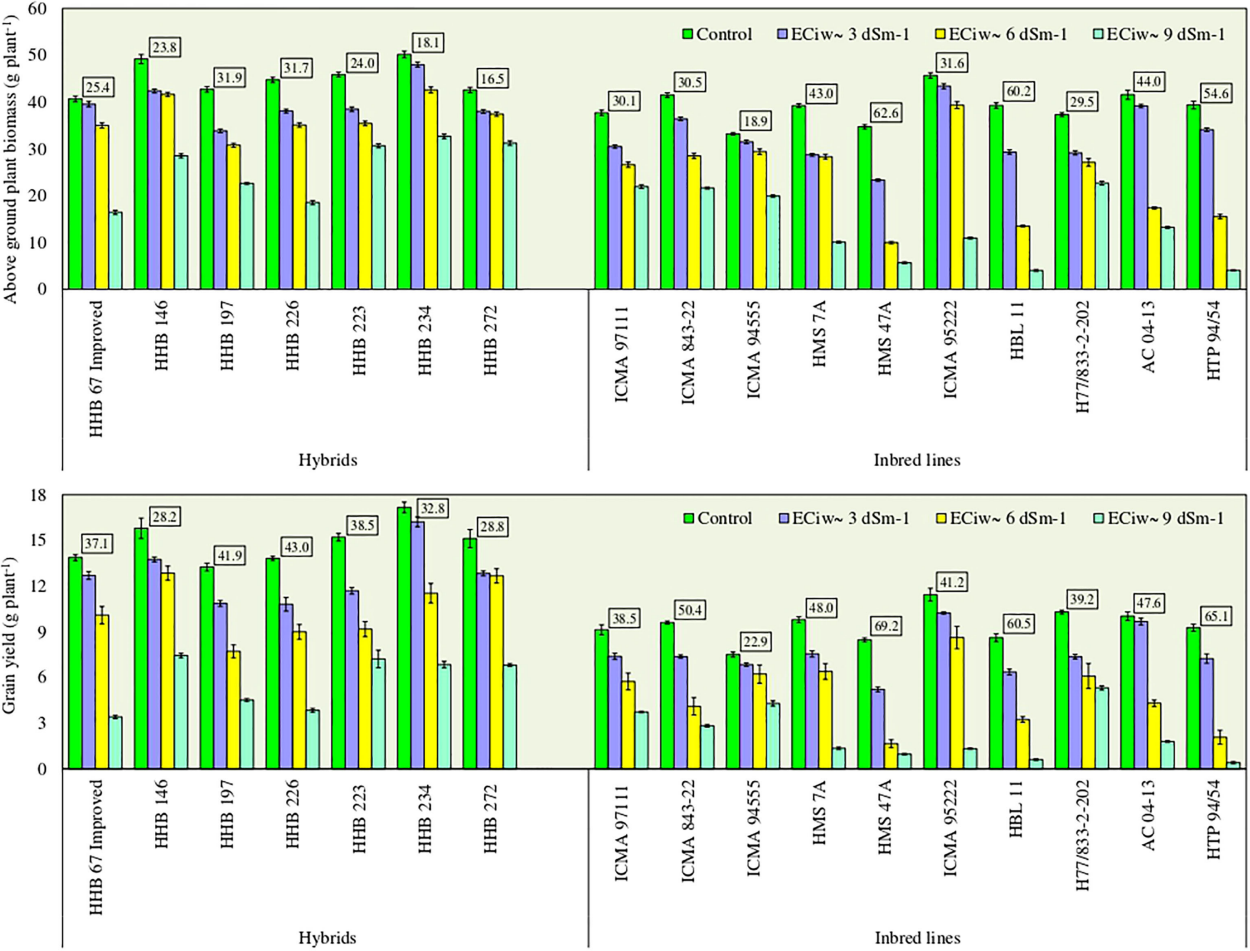
Effect of irrigation induced salinity stress on above ground plant biomass (AGB; g plant^-1^) and grain yield (GY; g plant^-1^) in pearl millet hybrids and inbred lines. Data represents mean value of 15 pooled measurements (3 plants per pot x 5 replications). Vertical bars labelled with boxes represent mean per cent reduction in AGB and GY due to salinity stress (averaged acrossed EC_iw_ of 3, 6 and 9 dS m^-1^) in comparison to control treatment receiving best available water (EC_iw_-0.6 dS m^-1^). Capped lines represent ± standard error of the mean values.

Plants exposure to salinity stress negatively affected the above ground biomass (AGB) accumulation. Compared to the control, the crop biomass production reduced by 17%, 40% and 65% across inbred lines, and 12%, 18% and 43% across hybrids with saline water irrigations of EC_iw_ ~3, 6 and 9 dSm^–1^, respectively (**Figure 1**). Five inbred lines (ICMA 94555, ICMA 95222, H7/822–2–202, HMS 7A and ICMA 97111) had biomass reduction upto 30% at EC_iw_ ~6 dSm^–1^, while this reduction stretched between 39–90% at higher salinity stress of EC_iw_ ~9 dSm^–1^. Within inbred lines, lowest biomass reduction was noticed for H77/833–2–202 (39%), followed by ICMA 94555 (40%), ICMA 97111 (42%) and ICMA 843–22 (48%). Most of the evaluated hybrids performed equally well upto EC_iw_ 6 dSm^–1^ attaining 31–43 g plant^–1^ dry biomass. Further increase in salinity stress had more pronounced effect on biomass reduction, except HHB 223, HHB 234 and HHB 272 for which <30% biomass reduction was noticed even when irrigated with EC_iw_ of 9 dSm^–1^ (**Figure 1**).

Experimental results indicated that the test genotypes displayed significant variability (p<0.0001) for grain yield in response to irrigation induced salinity stress (**Figure 1**). On an average, substantial yield reductions to the tune of 18%, 41% and 70% with saline water irrigations of EC_iw_ ~3, 6 and 9 dSm^–1^, respectively were observed; albeit to a greater extent in inbred lines compared to hybrids. On an average, hybrids produced 10.8–16.2 g plant^–1^ grain yield at EC_iw_ ~3 dSm^–1^, 7.7–12.9 g plant^–1^ at EC_iw_ ~6 dSm^–1^ and 3.4–7.4 g plant^–1^ at EC_iw_ ~9 dSm^–1^ (**Figure 1**). At higher salinity stress (EC_iw_ ~9 dSm^–1^), HHB 146 produced the highest grain yield (7.4 g plant^-1^) followed by HHB 223 (7.2 g plant^-1^), HHB 272 and HHB 234 (6.8 g plant^-1^) More importantly, the proportionate yield reductions remained <40% in the sequence of HHB 223 (39%) <HHB 234 (33%) <HHB 272 (29%) <HHB 146 (28%) when compared with their yields obtained at control. Conversely, inbred lines displayed higher yield reductions of 20% at EC_iw_ ~3 dSm^–1^, 49% at EC_iw_ ~6 dSm^–1^ and 76% at EC_iw_ ~9 dSm^–1^ (**Figure 1**). Across inbred lines, grain yield ranging between 1.7–8.6 g plant^–1^ with mean yield of 4.8 g plant^–1^ at EC_iw_ ~6 dSm^–1^ and 0.4–5.3 g plant^–1^ with mean yield of 2.3 g plant^–1^ at EC_iw_ ~9 dSm^–1^ was recorded. Similar to biomass trend, lowest yield reduction was noticed in ICMA 94555, followed by ICMA 97111, H77/833–2–202 and ICMA 95222.

### 3.6 Biplot analysis

**Figure 2:**
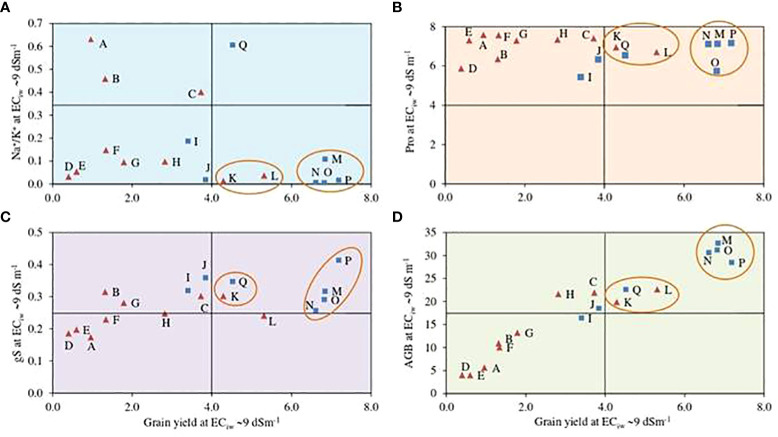
Biplots representing the interaction of important physiological traits; **(A)** sodium to potassium (Na^+^/K^+^) ratio, **(B)** proline content (Pro; mg g^-1^), **(D)**above ground biomass (AGB; g plant^-1^) and **(C)** stomatal condcutance (gS; mol m^-2^ s^-1^) with grain yield (GY; g plant^-1^) of pearl millet hybrids (blue ☐) and inbred lines (red ∆) at EC_iw_ -99 dSm^-1^. Data represents mean value of 15 pooled measurements (3 plants per pot x 5 replications); A: HMS 47A; B: ICMA 95222; C: ICMA 971ll; D: HTP 94/54; E: HBL 11; F: HMS 7A; G: AC04-13; H: ICMA 843-22; I: ICMA 94555; J: H77/833-2-202; K: HHB 67 Improved; L: HHB 226; M: HHB 234; N: HHB 223; O: HHB 272; P: HHB 146; Q: HHB 197.

A biplot between estimated grain yields and Na^+^/K^+^ ratio at EC_iw_ 9 dSm^–1^ illustrated that 4 pearl millet hybrids HHB 234, HHB 272, HHB 223 and HHB 146 exhibited better crop performance with low Na^+^/K^+^ accumulation in comparison to others (**Figure 2**); indicating their better adapatability and ion homeostasis in response to salinity stress. Similarly, only 2 inbred lines H77/833–2–202 and ICMA 94555 performed equally well at EC_iw_ 9 dSm^–1^ with yield ranging from 4–6 g plant^–1^ and lower Na^+^/K^+^ ratio (**Figure 2**). Osmolyte accumulation measured in terms of proline content showed that pearl millet hybrids HHB 234, HHB 272, HHB 223, HHB 197 and HHB 146, and inbred lines H77/833–2–202 and ICMA 94555 had higher osmolyte accumulation under higher salinity stress (**Figure 2**). Seven genotypes; including 2 inbred lines (H77/833–2–202 and ICMA 94555) and 5 hybrids (HHB 146, HHB 226, HHB 272, HHB 197 and HHB 67 Improved) confirmed their better adaptation to saline conditions showing relatively better stomatal conductance and yield performance at higher salinity stress (**Figure 2**). For biomass accumulation, a key fodder trait; 5 hybrids (HHB 146, HHB 197, HHB 223, HHB 234, HHB 272) and 2 inbred lines (ICMA 94555 and H77/833–2–202) showed their superiority producing higher biomass yield even at higher salinity stress of EC_iw_ ~9 dSm^–1^ (**Figure 2**).

### 3.7 Physiological traits association and trait modeling for higher grain yield under salinity stress

**Figure 3:**
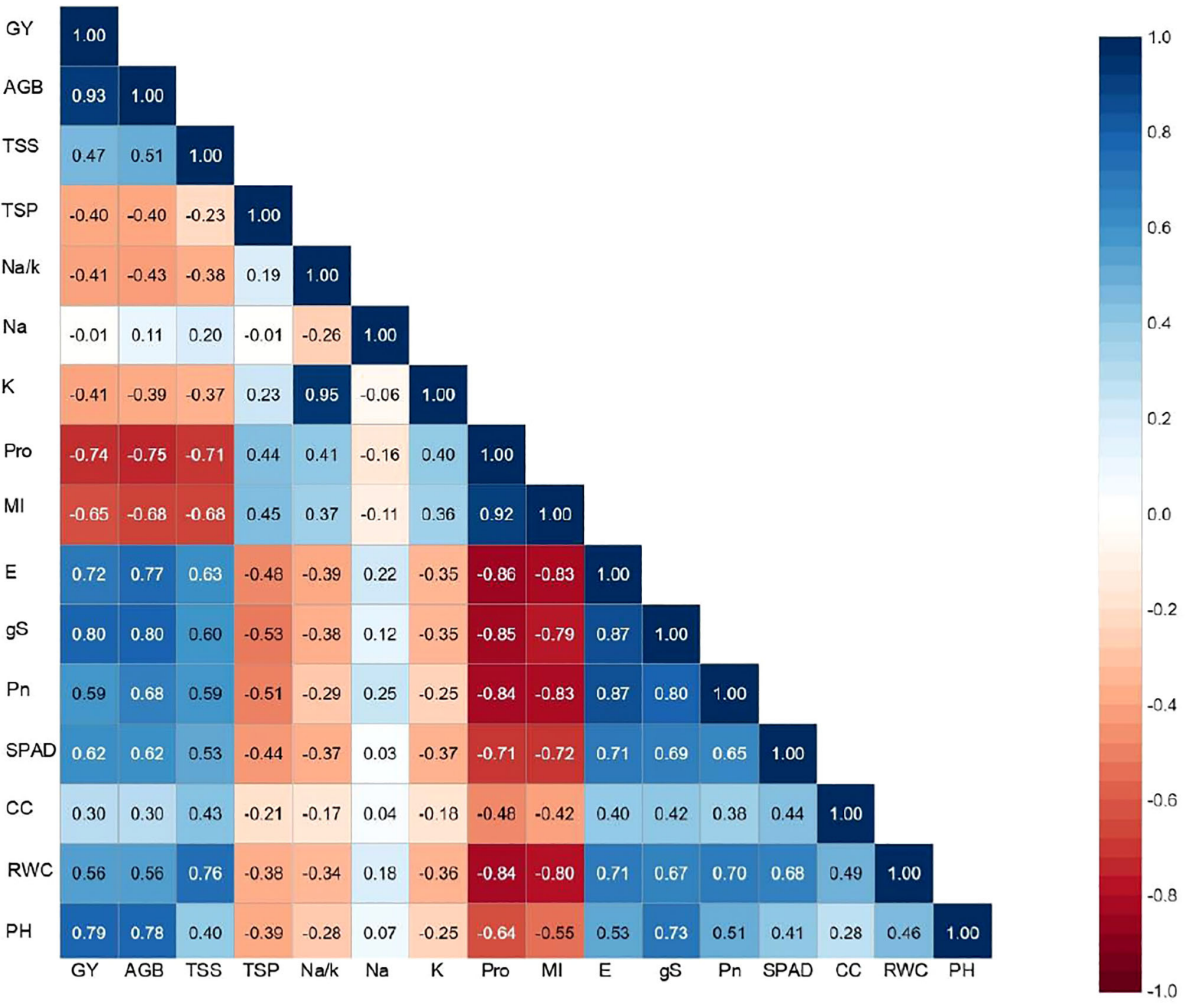
Traits association among morpho-physiological and yield parameters under irrigation induced salinity stress in pearl millet (averaged across evaluated hybrids and inbred lines). PH, plat height; RWC, relative water content; MI, membrane injury; CC; chlorophyll content; SPAD, soil plant analysis development (SPAD) chlorophyll meter reading; Pn, photosynthetic rate; gS, stomatal conductance; E, transpiration rate; Pro, proline; SP, soluble proteins; SS, soluble sugars; Na, sodium content; K, potassium content; Na/K, sodium to potassium ratio; AGB, above ground biomass.

Correlation matrix showing the association between different morpho-physiological traits of interest and the final output revealed a positive association of pearl millet yield with most of the evaluated parameters except for Na^+^, Na^+^/K^+^, Pro, MI and TSP (**Figure 3**). A strong and significant correlation was noticed between grain yield with AGB (0.93**), gS and E (>0.70**) and Pro (–0.74**) reflecting the influence of trait associated adaptation strategies to induced salinity stress, and their confounding effect on crop harvest. It was interesting to note a strong association between Na^+^ and Na^+^/K^+^ (0.95**), Pn, gS and E (>0.80**), RWC and MI (–0.80**) indicating a strong inter–dependence among physiological parameters of crop growth. Furthermore, a negative association of all the physiological traits was observed with shoot Na^+^ except MI and Pro which further increased with increasing levels of salinity. Inclusively, the trait association analysis revealed that most of the physiological traits were directly related to yield, and any deviation/disturbance in these traits led to decline in corresponding yield. The similar pattern of inter-trait associations were observed in inbreeds as well in hybrids except potassium content (K), which is significantly associated with the studied traits in inbreeds lines, but not in case of hybrids (**Supplementary Table 3**). Furthermore, all the studied traits (RWC, CC, SPAD, Pn, gS, E, MI, Pro, Na, K, Na/K, PRT, TS, BM) showed significant association with grain yield (Y) in control condition in both hybrids and inbreed lines, however, in salinity stress only seven physiological traits i.e. RWC, gS, E, Pro, Na, Na/K and BM were significantly associated (**Supplementary Table 4**).

To select the model physiological traits contributing maximum towards grain yield variations at higher salinity stress (EC_iw_ ~9 dSm^–1^), a stepwise regression approach (backward selection) was performed (**Supplementary Table 5**). The regression analysis indicated that a total of 7 traits (AGB, Pro, TSS, gS, SPAD, Pn, and TSP) in hybrids and 8 traits (AGB, Pro, PH, Na^+^, K^+^, Na^+^/K^+^, SPAD, and gS) in inbred lines significantly contributed towards grain yield variations in pearl millet (**Table 5**; **Supplementary Table 6**). It was interesting to note that above ground biomass (AGB) alone could justify >91% of grain yield variation in hybrids and inbreed lines at EC_iw_ ~9 dSm^–1^. Explicator traits such as Na^+^, K^+^ and Na^+^/K^+^ could only be utilized for the screening of inbred lines while AGB, Pro, gS and SPAD have higher weightage for pearl millet genotypes (inbred/hybrid) screening.

**Table 5:** Traits modeling for salinity tolerance in pearl millet through multiple linear regressions approach.

Dependent Variable	Steps and Variables	C(p)	R^2^-value	Adjusted R^2^-value
	Hybrids			
GY	AGB	140.412	91.46	91.35
	Pro + AGB	51.497	94.98	94.86
	Pro + TSS + AGB	30.916	95.86	95.70
	gS + Pro + TSS + AGB	14.941	96.55	96.38
	SPAD + gS + Pro + TSS + AGB	9.538	96.84	96.64
	SPAD + Pn + gS + Pro + TSS + AGB	7.772	96.99	96.75
	SPAD + Pn + gS + Pro + TSP + TSS + AGB	8.000	97.05	96.78
	Inbred lines			
GY	AGB	68.358	94.10	94.05
	Pro + AGB	49.566	94.77	94.68
	PH + Na^+^ + AGB	33.343	95.35	95.23
	Na^+^ + K^+^ + Na^+^/K^+^ + AGB	26.000	95.65	95.50
	PH + Na^+^ + K^+^ + Na^+^/K^+^ + AGB	11.693	96.17	96.00
	PH + SPAD + Na^+^ + K^+^ + Na^+^/K^+^ + AGB	8.396	96.34	96.15
	PH + SPAD + gS + Na^+^ + K^+^ + Na^+^/K^+^ + AGB	8.997	96.39	96.16
	PH + SPAD + gS + Pro + Na^+^ + K^+^ + Na^+^/K^+^ + AGB	9.000	96.45	96.19

Mallows’ Cp Criterionis a way to assess the fit of a multiple regression model; Smaller Cp values are better as they indicate smaller amounts of unexplained error; GY, grain yield; AGB, above ground biomass; Pro, proline content; TSP, total soluble protein; TSS, total soluble sugars; PH, plant height; Na^+^, sodium content; K^+^, potassium content; Na^+^/K^+^, sodium to potassium ratio; SPAD, soil plant analysis development (SPAD)chlorophyll meter reading; gS, Stomatal conductance.

### 3.8 Genotypic ranking for salinity tolerance

With the help of estimated regression coefficients of resp nbred lines and hybrids.


$$\text{Grain yield for inbred lines}=-6.56+0.014\times \text{PH}+0.031\times \text{SPAD}+3.418\times \text{gS}+0.156\times \text{Pro}+\left(-3.598\right)\times {\text{Na}}^{+}+0.288\times {\text{K}}^{+}+12.065\times {\text{Na}}^{+}/{\text{K}}^{+}+0.222\times \text{AGB}$$

Grain yield for hybrids=−1.520+(-0.050)×SPAD+0.050×Pn+8.750×gS+(-0.440)×Pro+0.050×TSP+(-0.180)×TSS+0.380×AGB

Based on predicted yields and resultant ranking, 3 pearl millet hybrids; HHB 146, HHB 272, and HHB 234 and 3 inbred lines; H77/833−2−202, ICMA 94555 and ICMA 843−22 had relatively higher ranking; suggesting that they would be more tolerant to irrigation induced salinity stress (**Supplementary Table 7A–D**). Conversely, HHB 226, HHB 67 Improved, and HHB 197 among evaluated hybrids, and HBL 11, HTP 94/54 and HMS 47A among inbred lines ranked lower and were found to be more sensitive to salt stress.

## 4 Discussion

The adverse effects of salinity and associated plant traits for tolerance have always been a researchable issue for plant scientists for development of better performing plant types. Herein, we evaluated the pearl millet hybrids and inbred lines for their response to saline irrigations, and identify key contributing traits for enhanced plant salt tolerance. In the present study, the genotypic differences within evaluated genotypes, and their consequent response to irrigation induced salinity stress led to alterations in plant morpho−physiological parameters of crop growth and their confounding effect on final harvest. Herein, the salinity induced reductions in plant height may be attributed to reduced osmotic pressure resulting in restricted water and nutrient uptake by the growing plants. Leaf RWC that generally represent the plant water status, declined substantially with stress mediated stomatal closure and restricted water loss from transpirational pathways compromising the leaf turgor (Polash et al., 2018). These variations in evaluated pearl millet genotypes could presumably be due to repressive effects of higher ion accumulation and hyper–osmotic stress on root hydraulic conductance and accelerated water loss from the leaf tissues (Dhansu et al., 2021; Sheoran et al., 2021). Earlier studies have also reported efficient water conservation system in pearl millet by means of lowering the leaf transpiration rate and reducing leaf area; hence, improved transpiration efficiency, plant water relations (RWC) and membrane stability under stress conditions (Vijayalakshmi et al., 2012; Reddy et al., 2022; Sheoran et al., 2022).

Salinity stress negatively affects both of the photosystems (PS I and PS II) and chlorophyll content (CC), owing to excessive accumulation of Na^+^ and Cl^–^ in the leaf tissues. This PS II mainly binds chlorophyll pigment for photosynthesis which tends to photo–damaged under stress conditions and hence, disturbs the metabolic pathways and enzyme activities responsible for synthesis/degradation of chlorophyll pigment (Kumar et al., 2018), thereby, decreasing chlorophyll content (Sneha et al., 2014). Chlorophyll meters are being used for monitoring leaf N status in agricultural crops in yield prediction, but the effects of environmental factors and leaf characteristics on leaf N estimations are still unclear. Xiong et al. (2015) observed a positive correlation between SPAD and chlorophyll content in different plant species including monocot and dicot species but the correlation of SPAD with total leaf nitrogen was different in two plant species of monocot and dicot. Our experimental findings also indicated that irrigation induced salinity stress significantly reduced the leaf gaseous exchange (Pn, gS and E), CC and SPAD values wherein the hybrids have higher photosynthetic efficiency than inbred lines. This could possibly be due to partial stomatal closure leading to reduction in intercellular CO_2_ concentration or chlorophyll degradation or reduced enzymatic activities or down regulation of proteins required to maintain structural integrity of photosystems (Kumar et al., 2016). Dudhate et al. (2018) also reported reduced photosynthesis due to decreased CC and SPAD values in pearl millet when exposed to drought stress. The genotypic differences and disturbed enzymes activities of ROS and photosynthetic pigments has also been reported earlier depicting their correlation with the presence or absence of a major terminal drought tolerance QTL (Kholova et al., 2011). The gene for chlorophyll a/b binding associated with both stay-green and grain yield traits under drought stress has been reported as a functional marker for selection of high yielding pearl millet genotypes with ‘stay green’ character under drought stress (Sehgal et al., 2015).

Exposure of plants to salinity stress triggers overproduction of ROS, which disrupts cell organelles and membrane components, inactivate enzyme, and also degrade protein complexes as well as nucleic acid (Tufail et al., 2018). Earlier reports have also shown the pronounced effects of salt stress on enhanced lipid peroxidation and protein oxidative damage, which in turn induces permeability impairment (Füzy et al., 2019). For osmotic adjustments, plants tend to accumulate compatible organic solutes (proline), soluble proteins and sugars for maintaining cellular homeostasis and osmoticum under saline conditions (Gharsallah et al., 2016). This could possibly be due to accumulation of low molecular weight proteins that might be utilized in the form of nitrogen during recovery process. Recently, putative WRKY protein factors have been identified in pearl millet in response to both dehydration and salinity stress involved in tolerance mechanisms (Chanwala et al., 2020). Upregulation of salt-induced proteins impart salt tolerance in tolerant pearl millet accessions with reduced expression in the sensitive accessions (Jha et al., 2022). Further, the sugars get accumulated under abiotic stress conditions due to decreased rate of respiration as well as down regulation of glycolysis (Nguyen et al., 2010).With increasing salinity stress, protective soluble proteins are synthesized *de novo* or may be present inheritably (Soni et al., 2020). Kusaka et al. (2005) and Ibrahimova et al. (2021) reported higher accumulation of osmolytes such as organic solutes (sucrose, glucose) and amino acids (proline) towards enhanced tolerance in pearl millet and wheat under stress conditions. Herein also, the osmolytes (Pro, TSP and TSS) were higher in inbred lines than pearl millet hybrids showing protective role of these osmolytes in better plant performance under salinity. This variability could have contributed towards their differential response in relative osmo–protectant and detoxification functions, and their role in buffering the cellular redox potential and protecting cellular structure under stress conditions (Sharma et al., 2021). Proteomic and physiological signatures also revealed the role of stress–related proteins in the root, mitochondrial electron transport, TCA cycle, C1–metabolism in leaf imparting stress tolerance in pearl millet (Ghatak et al., 2021). These genetic variations for proline accumulation in inbred lines could be ascribed to *de novo* synthesis or decrease in degradation of P5CS activity that allows favorable osmotic adjustments to regulate the adverse effects of salt stress (Sharma et al., 2014). Similar reports on positive interaction of osmotic stress, drought, and cold stress on proline synthesizing enzyme, P5CS1 was identified through network analysis in wheat through proline accumulation highlighting its protective role under abiotic stress conditions (Maghsoudi et al., 2019).

Ion homeostasis in a plant cell ensures its growth and development in normal environments as well as under unfavorable conditions through the absorption and compartmentalization of ions. Low levels of Na^+^/K^+^ ratio along with reduced Na^+^ and Cl^–^ loadings into the xylem is one of the major factor for normal functioning of the plant cell under stress conditions (Sharma et al., 2021).Similarly, K^+^ plays a key role in a myriad of physiological functions; protein synthesis, stomata opening and closing, phloem sugar loading and also acts as an organic osmolyte. Under salt stress, equilibrium status of Na/K plays an important role in balancing the ion toxicity in the cell. Previous studies have also documented the repressive effects of salinity stress on ionic imbalances in different crops including pearl millet (Venkata et al., 2012; Makarana et al., 2019a). This could be ascribed to restricted entry of Na^+^ into the leaf tissues and/or compartmentalization of Na^+^ either into the vacuoles or in stem portion. Since plants tend to accumulate toxic Na^+^ at the expense of essential K^+^ under salt stress; hence, favorable Na^+^/K^+^ ratio is a key indicator to visualize the stress associated plants behavior in maintaining ion balance. Our experimental evidences also highlighted the relevance of genotypic differences for salt tolerance in pearl millet by modulating favorable ionic balance through improved Na^+^ discrimination and preferential K^+^ uptake. We also observed that pearlmillet inbred lines maintained lower Na/K ratio than the hybrids by accumulating more K over Na ions (**Table 4**). Further, inbred lines AC 04-13 and ICMA 94555 and hybrids, HHB 223 and HHB 67 had higher K uptake than other plant types contributing towards better ion homeostasis and hence, salt tolerance. Chakraborty et al. (2022) also identified stress responsive genes in pearl millet inbred lines corresponding to ion and osmotic homeostasis, signal transduction, physiological adaptation and detoxification.

In our study, higher level of salinity stress (EC_iw_ ~9 dSm^–1^) induced an immediate and substantial adverse effect on plant growth and biomass accumulation, whereas moderate stress (EC_iw_ ~3 and 6 dSm^−1^) compensated in cumulative response compared to the control plants. Higher biomass production under these conditions might be due to accumulation of inorganic ions and compatible organic solutes for osmotic adjustments. A significant positive correlation of yield with leaf water potential, relative water content, stomatal conductance, photosynthetic rate, proline, total soluble sugars, free amino acids, membrane stability index, leaf area index and total biomass under water-deficit stress (Vijayalakshmi et al., 2012) and salt stress (Makarana et al., 2019b) has been reported earlier in pearl millet A positive correlation of MDA content and proline has been reported with accumulation of green and dry biomass in best-performing pearl millet lines under ionic stress (Toderich et al., 2018).Association analysis of a total of 392,493 SNPs identified QTLs for biomass production in early drought stress conditions and for stay-green trait in pearlmillet using Genotyping-by-Sequencing (GBS) (Debieu et al., 2018). In our studies, the comparative analysis of association of physiological traits between pearl millet hybrids and inbred lines indicated a parallel trait association among the two although the K^+^ ion uptake discriminated the two (**Supplementary Table 3**). As the SPAD reading (indicative of leaf greenness) increases total chlorophyll content also increased in both hybrids and inbreeds with higher magnitude in hybrids. Because, abiotic stress tend to reduce leaf area and hence, concentrate of leaf pigments. Consequently, higher photosynthetic rate in inbred lines than the hybrids was observed in our experiments. Further, at higher salinity level, accumulation of higher proline and total proteins have a negative association with the total soluble sugars in hybrids than the inbred lines.

In finger millet (*Eleusine coracana*), the sensitivity of the growth stage towards drought stress was indicated through biplot analysis along with significant genotypic variation (Mude et al., 2020). Contrarily, the reduction in biomass may be linked to restricted hydrolysis of reserved foods with limited nutrient uptake and their translocation to the growing plant parts (Yamazaki et al., 2020).

Grain yield formation in plants depends entirely on the ability of the crop plants to assimilate and utilize the available growth resources, and thus, is an interplay of several cellular and functional components contributing towards final harvest. Ghatak et al. (2021) explored physiological and proteomic signatures for drought resilience and observed maintenance of pearl millet and wheat grain yield under drought stress. Identification of an important SNP in putative acetyl CoA carboxylase gene has showed significant association with grain yield, grain harvest index and panicle yield under drought stress in pearl millet (Sehgal et al. (2015). Generally, all the glycophytes show yield reductions under saline conditions owing to disturbed water and nutritional balance, decreased source to sink ratio and poor plant photosynthetic efficiency (Yadav et al., 2020). Further, reduction in the grain yield might possibly be due to decreased pollen viability, stigma receptivity, poor seed setting and reduced seed weight under saline environments that ultimately culminate in lower crop yields (Sharma et al., 2021). In this study, increasing salinity stress might have restricted the availability of growth resources for plant survival and hindered photosynthetic activity exposing them to deficient minerals nutrition and water uptake and ultimately reduced the crop (Toderich et al., 2018; Yadav et al., 2020). Recently, genome–wide association (GWAS) and genomic prediction for improving drought stress tolerance in pearl millet revealed high prediction accuracy and heritability between yield–associated traits and hybrid performance across different drought prone growing environments (Varshney et al., 2017). More importantly, the prediction of hybrid performance through genomic selection strategy with additive and dominance effects identified 159 combinations which have never been used in breeding programme and therefore, these were proposed as good candidates for development of high–yielding pearl millet hybrids.

## 5 Conclusions

The performance of pearl millet hybrids and inbred lines assessed through traits modeling approach helped to identify key morpho–physiological traits governing the anticipated salt tolerance, plant adaptation and grain yield variations in response to irrigation induced salinity stress. The plant functioning traits like higher photosynthetic rate, lower Na^+^/K^+^ ratio and higher biomass accumulation could be effectively utilized for screening and identification of potential salt tolerant pearl millet germplasm. The experimental findings revealed that the pearl millet hybrids; HHB 146, HHB 272, HHB 234 and the inbred lines; H77/833–2–202, ICMA 94555 and ICMA 843–22, showed trait-associated better adaptation mechanisms and perceived lesser yield reduction with increasing salinity stress. These pearl millet hybrids could be recommended for enhancing the crop resilience, stabilize production and generate higher income in saline agro−ecosystems. More importantly, the identified inbred lines with special characteristics (salt tolerance) may be utilized as potential genetic source in pearl millet developmental program for salt−affected ecologies. Recent advancements of biotechnological and genomic tools like genome-wide SNPs mining through genome sequencing and resequencing in pearl millet breeding are being applied as in other important crops, which will further facilitate the efforts for mapping of complex, polygenic controlled important traits, such as abiotic stress tolerance (salinity, drought and heat), yield contributing traits and will speed up the pearl millet improvement program.

## Data availability statement

The original contributions presented in the study are included in the article/**Supplementary Material**. Further inquiries can be directed to the corresponding authors.

## Author contributions

AsK and PS: investigation, data visualization, original draft preparation; AM, DY and DKS: conceptualization and final editing; ArK: statistical analysis, SD: original draft preparation, NK and PD: data visualization. All authors contributed to the article and approved the submitted version.
