# Supplementary material for: Deciphering trait associated morpho-physiological responses in pearlmillet hybrids and inbred lines under salt stress
Source: Front Plant Sci. 2023 Mar 2;14:1121805. doi: 10.3389/fpls.2023.1121805 (PMC10018183; doi:10.3389/fpls.2023.1121805)
Supplement: Supplementary file 1 [file DataSheet_1.docx]

**SUPPLEMENTARY FIGURE 1. Status of irrigation induced salinity buildup EC_e_ (dSm^–1^) in soil at different stages.**

**
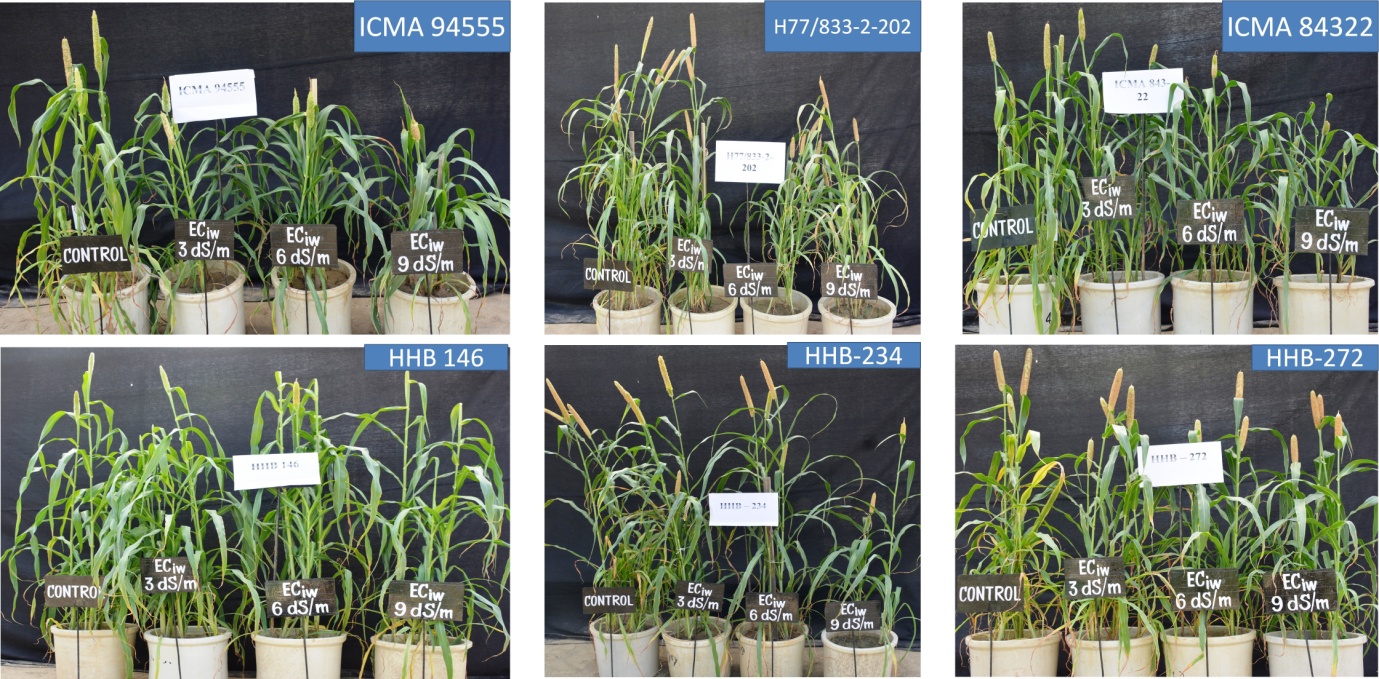
**

**SUPPLEMENTARY FIGURE 2. Effect of salinity stress on best performing pearl millet**

**inbred lines and hybrids.**

**SUPPLEMENTARY TABLE 1. Brief description of evaluated pearl millet hybrids and inbred lines.**

| **S. No.** | **Name** | **Pedigree / Parentage** | **Year of release** | **Characteristic features** |
| --- | --- | --- | --- | --- |
| **Hybrids** | | | | |
| 1. | HHB 67 Improved | ICMA 843-22 × H77/833-2-202 | 2005 | Rainfed ecosystems, suitable for early/late planting, 62-65 days maturity, highly resistant to downy mildew, tolerant to drought, good tillering |
| 2. | HHB 146 | ICMA 95222 × HTP 94/54 | 2003 | Irrigated ecosystems, 75-80 days maturity, resistant to downy mildew and fairly tolerant to drought |
| 3. | HHB 197 | ICMA 97111 × HBL 11 | 2008 | Rainfed/irrigated ecosystems, 68-72 days maturity, highly resistant to downy mildew, tolerant to drought |
| 4. | HHB 226 | ICMA 843-22 × HBL 11 | 2011 | Rainfed ecosystems, 70-72 days maturity, resistant to downy mildew |
| 5. | HHB 223 | ICMA 94555 × HBL 11 | 2010 | Rainfed/semi-irrigated ecosystems, 70-75 days maturity, resistant to downy mildew, tolerant to drought |
| 6. | HHB 234 | HMS 7A × H77/833-2-202 | 2013 | Rainfed ecosystems, 70-75 days maturity, resistant to downy mildew and blast, thin stem and high tillering |
| 7. | HHB 272 | HMS 47A × AC 04/13 | 2016 | Rainfed ecosystems, 65-68 days maturity, resistant to downy mildew and blast |
| **Inbred lines** | | | | |
| 8. | ICMA 97111 | ICMB 97111 backcrossed to 863 A cytoplasm (A_1_) source | 1997 | Female parent of HHB 197, high tillering |
| 9. | ICMA 843-22 | BKM 2068 backcrossed to AKM 2068 cytoplasm (A_1_) source | - | Female parent of HHB 67 IMP and HHB 226, early, D_2_ dwarf, downy mildew resistant and high tillering |
| 10. | ICMA 94555 | ICMB 94555 backcrossed to 81A cytoplasm (A_1_) source | 1994 | Female parent of HHB 223, early flowering, D_2_ dwarf |
| 11. | HMS 7A | HMS 7B backcrossed to 81A cytoplasm (A_1_) source | 1990 | Female parent of HHB 234 |
| 12. | HMS 47A | HMS 47B backcrossed to HMS 18A cytoplasm (A_1_) source | 2004 | Female parent of HHB 272 |
| 13. | ICMA 95222 | ICMB 95222 backcrossed to 81A cytoplasm (A_1_) source | 1995 | Female parent of HHB 146, medium to late flowering |
| 14. | HBL 11 | R line of A_1_ cytoplasm | - | Male parent of hybrids HHB 197, HHB 223 and HHB 226 |
| 15. | H77/833-2-202 | R line of A_1_ cytoplasm | - | Male parent of HHB 67IMP and HHB 234, very early flowering, good tillering, thin stem |
| 16. | AC 04-13 | R line of A_1_ cytoplasm | - | Male parent of HHB 272, early to medium flowering, good tillering |
| 17. | HTP 94/54 | R line of A_1_ cytoplasm | - | Male parent of HHB 146, medium to late flowering |

**SUPPLEMENTARY TABLE 2: Test of normality of variables and transformation methods**

| **Variable** | REP | **Testing Method** | **W Value** | **Pr(<W)** | **Transformation** |
| --- | --- | --- | --- | --- | --- |
| PH | 1 | Shapiro-Wilk | 0.917 | 0.0002 | **log Y** |
| PH | 2 | Shapiro-Wilk | 0.9197 | 0.0003 | **log Y** |
| PH | 3 | Shapiro-Wilk | 0.9281 | 0.0007 | **log Y** |
| RWC | 1 | Shapiro-Wilk | 0.9529 | 0.0119 | **log Y** |
| RWC | 2 | Shapiro-Wilk | 0.9543 | 0.0141 | **log Y** |
| RWC | 3 | Shapiro-Wilk | 0.954 | 0.0136 | **log Y** |
| CC | 1 | Shapiro-Wilk | 0.9739 | **0.1650** | **NO** |
| CC | 2 | Shapiro-Wilk | 0.9688 | **0.0856** | **NO** |
| CC | 3 | Shapiro-Wilk | 0.8972 | 0.0000 | **log (Y+1)** |
| SPAD | 1 | Shapiro-Wilk | 0.9851 | **0.5925** | **NO** |
| SPAD | 2 | Shapiro-Wilk | 0.9856 | **0.6200** | **NO** |
| SPAD | 3 | Shapiro-Wilk | 0.9827 | **0.4642** | **NO** |
| Pn | 1 | Shapiro-Wilk | 0.9496 | 0.0080 | **log Y** |
| Pn | 2 | Shapiro-Wilk | 0.9535 | 0.0128 | **log Y** |
| Pn | 3 | Shapiro-Wilk | 0.9595 | 0.0266 | **log Y** |
| gS | 1 | Shapiro-Wilk | 0.9749 | **0.1868** | **NO** |
| gS | 2 | Shapiro-Wilk | 0.9799 | **0.3404** | **NO** |
| gS | 3 | Shapiro-Wilk | 0.9816 | **0.4134** | **NO** |
| E | 1 | Shapiro-Wilk | 0.9689 | **0.0866** | **NO** |
| E | 2 | Shapiro-Wilk | 0.9687 | **0.0848** | **NO** |
| E | 3 | Shapiro-Wilk | 0.9622 | 0.0372 | **log (Y+1)** |
| MI | 1 | Shapiro-Wilk | 0.9021 | 0.0001 | **log (Y+1)** |
| MI | 2 | Shapiro-Wilk | 0.9054 | 0.0001 | **log (Y+1)** |
| MI | 3 | Shapiro-Wilk | 0.9067 | 0.0001 | **log (Y+1)** |
| Pro | 1 | Shapiro-Wilk | 0.9094 | 0.0001 | **log (Y+1)** |
| Pro | 2 | Shapiro-Wilk | 0.9102 | 0.0001 | **log (Y+1)** |
| Pro | 3 | Shapiro-Wilk | 0.9169 | 0.0002 | **log (Y+1)** |
| Na | 1 | Shapiro-Wilk | 0.653 | 0.0000 | **log (Y+1)** |
| Na | 2 | Shapiro-Wilk | 0.6576 | 0.0000 | **log (Y+1)** |
| Na | 3 | Shapiro-Wilk | 0.6545 | 0.0000 | **log (Y+1)** |
| K | 1 | Shapiro-Wilk | 0.8977 | 0.0000 | **log (Y+1)** |
| K | 2 | Shapiro-Wilk | 0.9098 | 0.0001 | **log (Y+1)** |
| K | 3 | Shapiro-Wilk | 0.9072 | 0.0001 | **log (Y+1)** |
| NaK | 1 | Shapiro-Wilk | 0.5601 | 0.0000 | **log (Y+1)** |
| NaK | 2 | Shapiro-Wilk | 0.5625 | 0.0000 | **log (Y+1)** |
| NaK | 3 | Shapiro-Wilk | 0.5558 | 0.0000 | **log (Y+1)** |
| PRT | 1 | Shapiro-Wilk | 0.9707 | **0.1097** | **NO** |
| PRT | 2 | Shapiro-Wilk | 0.9708 | **0.1114** | **NO** |
| PRT | 3 | Shapiro-Wilk | 0.9826 | **0.4619** | **NO** |
| TS | 1 | Shapiro-Wilk | 0.9728 | **0.1429** | **NO** |
| TS | 2 | Shapiro-Wilk | 0.9755 | **0.2014** | **NO** |
| TS | 3 | Shapiro-Wilk | 0.9807 | **0.3718** | **NO** |
| BM | 1 | Shapiro-Wilk | 0.9451 | 0.0048 | **log Y** |
| BM | 2 | Shapiro-Wilk | 0.9443 | 0.0043 | **log Y** |
| BM | 3 | Shapiro-Wilk | 0.9473 | 0.0061 | **log Y** |
| Y | 1 | Shapiro-Wilk | 0.9799 | **0.3392** | **NO** |
| Y | 2 | Shapiro-Wilk | 0.9808 | **0.3775** | **NO** |
| Y | 3 | Shapiro-Wilk | 0.98 | **0.3458** | **NO** |

**SUPPLEMENTARY TABLE 3: Comparative association of physiological traits in pearl millet hybrids and inbreeds**

|  |  | **RWC** | **CC** | **SPAD** | **Pn** | **gS** | **E** | **MI** | **Pro** | **Na** | **K** | **NaK** | **PRT** | **TS** | **BM** | **Y** |
| --- | --- | --- | --- | --- | --- | --- | --- | --- | --- | --- | --- | --- | --- | --- | --- | --- |
| **PH** | **Hybrids** | 0.519^**^ | 0.360^**^ | 0.495^**^ | 0.532^**^ | 0.749^**^ | 0.608^**^ | -0.554^**^ | -0.648^**^ | -0.248^**^ | -0.025 | -0.279^**^ | -0.403^**^ | 0.503^**^ | 0.806^**^ | 0.800^**^ |
|  | **Inbreed** | 0.614^**^ | 0.363^**^ | 0.437^**^ | 0.651^**^ | 0.723^**^ | 0.548^**^ | -0.700^**^ | -0.738^**^ | -0.224^*^ | 0.344^**^ | -0.286^**^ | -0.410^**^ | 0.418^**^ | 0.728^**^ | 0.758^**^ |
| **RWC** | **Hybrids** |  | 0.487^**^ | 0.716^**^ | 0.703^**^ | 0.692^**^ | 0.763^**^ | -0.819^**^ | -0.844^**^ | -0.403^**^ | **-0.022** | -0.367^**^ | -0.467^**^ | 0.750^**^ | 0.646^**^ | 0.683^**^ |
|  | **Inbreed** |  | 0.546^**^ | 0.600^**^ | 0.703^**^ | 0.733^**^ | 0.660^**^ | -0.764^**^ | -0.852^**^ | -0.320^**^ | 0.381^**^ | -0.326^**^ | -0.270^**^ | 0.803^**^ | 0.598^**^ | 0.644^**^ |
| **CC** | **Hybrids** |  |  | 0.529^**^ | 0.458^**^ | 0.496^**^ | 0.482^**^ | -0.422^**^ | -0.529^**^ | -0.209^*^ | -0.075 | -0.196^*^ | -0.277^**^ | 0.434^**^ | 0.393^**^ | 0.411^**^ |
|  | **Inbreed** |  |  | 0.384^**^ | 0.354^**^ | 0.430^**^ | 0.374^**^ | -0.447^**^ | -0.485^**^ | -0.200^*^ | 0.177 | -0.187^*^ | -0.184^*^ | 0.457^**^ | 0.328^**^ | 0.364^**^ |
| **SPAD** | **Hybrids** |  |  |  | 0.622^**^ | 0.731^**^ | 0.728^**^ | -0.724^**^ | -0.756^**^ | -0.323^**^ | -0.167 | -0.310^**^ | -0.508^**^ | 0.576^**^ | 0.680^**^ | 0.715^**^ |
|  | **Inbreed** |  |  |  | 0.684^**^ | 0.668^**^ | 0.705^**^ | -0.680^**^ | -0.635^**^ | -0.448^**^ | **0.255^**^** | -0.483^**^ | -0.328^**^ | 0.499^**^ | 0.623^**^ | 0.648^**^ |
| **Pn** | **Hybrids** |  |  |  |  | 0.762^**^ | 0.848^**^ | -0.805^**^ | -0.832^**^ | -0.176^*^ | 0.093 | -0.200^*^ | -0.526^**^ | 0.648^**^ | 0.617^**^ | 0.594^**^ |
|  | **Inbreed** |  |  |  |  | 0.900^**^ | 0.891^**^ | -0.848^**^ | -0.862^**^ | -0.363^**^ | **0.388^**^** | -0.405^**^ | -0.499^**^ | 0.557^**^ | 0.876^**^ | 0.875^**^ |
| **gS** | **Hybrids** |  |  |  |  |  | 0.866^**^ | -0.771^**^ | -0.835^**^ | -0.316^**^ | -0.063 | -0.323^**^ | -0.596^**^ | 0.651^**^ | 0.753^**^ | 0.795^**^ |
|  | **Inbreed** |  |  |  |  |  | 0.900^**^ | -0.851^**^ | -0.876^**^ | -0.381^**^ | **0.397^**^** | -0.432^**^ | -0.428^**^ | 0.588^**^ | 0.844^**^ | 0.862^**^ |
| **E** | **Hybrids** |  |  |  |  |  |  | -0.862^**^ | -0.890^**^ | -0.303^**^ | 0.044 | -0.324^**^ | -0.545^**^ | 0.705^**^ | 0.759^**^ | 0.764^**^ |
|  | **Inbreed** |  |  |  |  |  |  | -0.779^**^ | -0.823^**^ | -0.406^**^ | **0.423^**^** | -0.466^**^ | -0.395^**^ | 0.550^**^ | 0.856^**^ | 0.840^**^ |
| **MI** | **Hybrids** |  |  |  |  |  |  |  | 0.912^**^ | 0.273^**^ | 0.001 | 0.271^**^ | 0.477^**^ | -0.706^**^ | -0.666^**^ | -0.683^**^ |
|  | **Inbreed** |  |  |  |  |  |  |  | 0.938^**^ | 0.502^**^ | **-0.217^*^** | 0.492^**^ | 0.405^**^ | -0.670^**^ | -0.793^**^ | -0.812^**^ |
| **Pro** | **Hybrids** |  |  |  |  |  |  |  |  | 0.357^**^ | -0.010 | 0.356^**^ | 0.500^**^ | -0.745^**^ | -0.738^**^ | -0.766^**^ |
|  | **Inbreed** |  |  |  |  |  |  |  |  | 0.468^**^ | **-0.350^**^** | 0.470^**^ | 0.363^**^ | -0.707^**^ | -0.824^**^ | -0.846^**^ |
| **Na** | **Hybrids** |  |  |  |  |  |  |  |  |  | 0.012 | 0.965^**^ | 0.236^**^ | -0.380^**^ | -0.391^**^ | -0.404^**^ |
|  | **Inbreed** |  |  |  |  |  |  |  |  |  | -0.188 | 0.940^**^ | 0.222^*^ | -0.372^**^ | -0.370^**^ | -0.431^**^ |
| **K** | **Hybrids** |  |  |  |  |  |  |  |  |  |  | **-0.170** | **0.068** | **0.052** | **-0.024** | **-0.132** |
|  | **Inbreed** |  |  |  |  |  |  |  |  |  |  | **-0.413^**^** | **-0.135** | **0.316^**^** | **0.367^**^** | **0.394^**^** |
| **NaK** | **Hybrids** |  |  |  |  |  |  |  |  |  |  |  | 0.203^*^ | -0.389^**^ | -0.415^**^ | -0.400^**^ |
|  | **Inbreed** |  |  |  |  |  |  |  |  |  |  |  | 0.171^*^ | -0.389^**^ | -0.437^**^ | -0.474^**^ |
| **PRT** | **Hybrids** |  |  |  |  |  |  |  |  |  |  |  |  | -0.373^**^ | -0.424^**^ | -0.443^**^ |
|  | **Inbreed** |  |  |  |  |  |  |  |  |  |  |  |  | -0.090 | -0.369^**^ | -0.367^**^ |
| **TS** | **Hybrids** |  |  |  |  |  |  |  |  |  |  |  |  |  | 0.583^**^ | 0.549^**^ |
|  | **Inbreed** |  |  |  |  |  |  |  |  |  |  |  |  |  | 0.500^**^ | 0.531^**^ |
| **BM** | **Hybrids** |  |  |  |  |  |  |  |  |  |  |  |  |  |  | 0.945^**^ |
|  | **Inbreed** |  |  |  |  |  |  |  |  |  |  |  |  |  |  | 0.970^**^ |

**SUPPLEMENTARY TABLE 4: Magnitude of association of physiological traits with grain yield**

| **Traits** | | **PH** | **RWC** | **CC** | **SPAD** | **Pn** | **gS** | **E** | **MI** | **Pro** | **Na** | **K** | **NaK** | **PRT** | **TS** | **BM** |
| --- | --- | --- | --- | --- | --- | --- | --- | --- | --- | --- | --- | --- | --- | --- | --- | --- |
| **YIELD** | **CONTROL** | .648** | .528** | .350** | .585** | .343** | .604** | .565** | -.586** | -.631** | -.326** | -.268** | -.293** | -.402** | .265** | .883** |
|  | **SALINITY** | .766** | -.298* | -0.121 | 0.101 | 0.059 | .718** | .362** | 0.219 | -.350** | -.351** | -0.08 | -.391** | -0.079 | -0.136 | .940** |

**SUPPLEMENTARY TABLE 5. Traits prioritization for salinity stress tolerance in pearl millet through regression analysis.**

| **Variables** | **Regression coefficient (β_s_)** | **Std. Error** | **t-value** | **Pr(>\|t\|)** |
| --- | --- | --- | --- | --- |
|  | **Hybrids** | | | |
| Intercept | -2.917 | 3.805 | -0.767 | 0.446 |
| PH* | -0.007 | 0.014 | -0.447 | 0.656 |
| RWC* | 0.050 | 0.026 | 1.881 | 0.064 |
| MI* | 0.006 | 0.028 | 0.197 | 0.845 |
| CC* | -0.302 | 0.234 | -1.292 | 0.201 |
| SPAD | -0.051 | 0.024 | -2.076 | 0.042 |
| Pn | 0.076 | 0.029 | 2.640 | 0.010 |
| gS | 11.923 | 2.561 | 4.655 | 0.000 |
| E* | -0.181 | 0.207 | -0.872 | 0.386 |
| Pro | -0.350 | 0.158 | -2.218 | 0.030 |
| TSP | 0.082 | 0.040 | 2.086 | 0.041 |
| TSS | -0.195 | 0.034 | -5.717 | 0.000 |
| Na^+^* | 5.955 | 4.183 | 1.424 | 0.159 |
| K^+^* | -0.485 | 0.276 | -1.757 | 0.083 |
| Na^+^/K^+^* | -28.973 | 19.424 | -1.492 | 0.140 |
| AGB | 0.373 | 0.023 | 16.022 | 0.000 |
|  | **Inbred lines** | | | |
| Intercept | -6.643 | 1.884 | -3.527 | 0.001 |
| PH | 0.012 | 0.004 | 3.164 | 0.002 |
| RWC* | 0.001 | 0.020 | 0.037 | 0.970 |
| MI* | -0.011 | 0.020 | -0.561 | 0.576 |
| CC* | 0.053 | 0.174 | 0.306 | 0.760 |
| SPAD | 0.030 | 0.015 | 1.980 | 0.050 |
| Pn* | 0.030 | 0.023 | 1.293 | 0.199 |
| gS | 3.840 | 1.840 | 2.087 | 0.039 |
| E* | -0.148 | 0.114 | -1.296 | 0.198 |
| Pro | 0.215 | 0.130 | 1.655 | 0.101 |
| TSP | 0.088 | 0.025 | 3.526 | 0.001 |
| TSS* | -0.003 | 0.016 | -0.213 | 0.832 |
| Na^+^ | -3.647 | 0.551 | -6.623 | 0.000 |
| K^+^ | 0.309 | 0.067 | 4.655 | 0.000 |
| Na^+^/K^+^ | 12.182 | 2.117 | 5.755 | 0.000 |
| AGB | 0.223 | 0.013 | 16.613 | 0.000 |

**Variables not considered in traits modeling; PH: plant height; RWC: relative water content; MI: membrane injury; CC: chlorophyll content, SPAD: soil plant analysis development (SPAD) chlorophyll meter reading; Pn: photosynthetic rate; gS: stomatal conductance; E: transpiration rate; Pro: proline; TSP: total soluble protein; TSS: total soluble sugars; Na^+^: sodium content; K^+^: potassium content; Na^+^/K^+^: sodium to potassium ratio; AGB: above ground biomass.*

**SUPPLEMENTARY TABLE 6. Regression coefficient, standard error, and significance of the prioritized traits for salinity stress tolerance in pearl millet.**

| **Dependent Variable** | **Variables** | **Regression coefficient (βs)** | **Std. Error** | **t-value** | **Pr(>\|t\|)** |
| --- | --- | --- | --- | --- | --- |
|  | **Hybrids** | | | | |
| GY | Intercept | -1.520 | 1.760 | -0.860 | 0.390 |
|  | PH | -0.050 | 0.020 | -2.450 | 0.017 |
|  | SPAD | 0.050 | 0.030 | 2.040 | 0.045 |
|  | gS | 8.750 | 1.980 | 4.420 | 0.000 |
|  | Pro | -0.440 | 0.100 | -4.360 | 0.000 |
|  | TSP | 0.050 | 0.040 | 1.330 | 0.187 |
|  | TSS | -0.180 | 0.030 | -5.760 | 0.000 |
|  | AGB | 0.380 | 0.020 | 24.420 | 0.000 |
|  | Model Fitted: GY~ -1.520+ (-0.050)*PH + 0.050*SPAD+ 8.750*gS + (-0.440)*Pro + 0.050*SP + (-0.180)*SS+ 0.380*AGB | | | | |
|  | **Inbred lines** | | | | |
| GY | Intercept | -6.643 | 1.884 | -3.527 | 0.001 |
|  | PH | 0.014 | 0.003 | 4.732 | 0.000 |
|  | SPAD | 0.031 | 0.013 | 2.411 | 0.018 |
|  | gS | 3.418 | 1.375 | 2.485 | 0.015 |
|  | Pro | 0.156 | 0.068 | 2.309 | 0.023 |
|  | Na^+^ | -3.598 | 0.528 | -6.815 | 0.000 |
|  | K^+^ | 0.288 | 0.056 | 5.133 | 0.000 |
|  | Na^+^/K^+^ | 12.065 | 1.983 | 6.085 | 0.000 |
|  | AGB | 0.222 | 0.010 | 22.982 | 0.000 |
|  | Model Fitted: GY~ -6.56 + 0.014*PH + 0.031*SPAD + 3.418*gS + 0.156*Pro + (-0.598)*Na^+^ + 0.288*K^+^ + 12.065*Na^+^/K^+^ + 0.222*AGB | | | | |

*GY; grain yield; PH: plant height; SPAD: soil plant analysis development (SPAD) chlorophyll meter reading; gS: stomatal conductance; Pro: proline; TSP: total soluble protein; TSS: total soluble sugars; Na^+^: sodium content; K^+^: potassium content; Na^+^/K^+^: sodium to potassium ratio; AGB: above ground biomass.*

**SUPPLEMENTARY TABLE 7a. Mean response of model physiological traits in pearl millet hybrids at higher salinity stress (EC_iw_ ~9 dSm^–1^).**

| **Hybrids** | **Traits** | | | | | | |
| --- | --- | --- | --- | --- | --- | --- | --- |
|  | **SPAD** | **Pn** | **gS** | **Pro** | **TSP** | **TSS** | **AGB** |
| HHB 67 Improved | 30.400 | 19.360 | 0.319 | 5.437 | 13.040 | 10.727 | 16.333 |
| HHB 146 | 39.033 | 15.400 | 0.414 | 7.170 | 10.237 | 11.123 | 28.333 |
| HHB 197 | 40.000 | 18.653 | 0.347 | 6.543 | 12.547 | 11.137 | 22.667 |
| HHB 226 | 31.567 | 12.920 | 0.359 | 6.337 | 11.530 | 15.417 | 18.333 |
| HHB 223 | 35.933 | 9.253 | 0.257 | 7.117 | 12.517 | 14.177 | 30.333 |
| HHB 234 | 43.167 | 7.760 | 0.317 | 7.130 | 12.053 | 12.327 | 32.333 |
| HHB 272 | 45.133 | 17.137 | 0.291 | 5.743 | 8.823 | 14.590 | 31.333 |

*SPAD: soil plant analysis development (SPAD) chlorophyll meter reading; Pn: photosynthetic rate; gS: stomatal conductance; Pro: proline; TSP: total soluble protein; SS: total soluble sugars; Na^+^: sodium content; K^+^: potassium content; Na^+^/K^+^: sodium to potassium ratio; AGB: above ground biomass.*

**SUPPLEMENTARY TABLE 7b. Mean response of model physiological traits in pearl millet inbred lines at higher salinity stress (EC_iw_ ~9 dSm^–1^).**

| **Inbred lines** | **Traits** | | | | | | | |
| --- | --- | --- | --- | --- | --- | --- | --- | --- |
|  | **PH** | **SPAD** | **gS** | **Pro** | **Na^+^** | **K^+^** | **Na^+^/K^+^** | **AGB** |
| ICMA 97111 | 114.003 | 46.200 | 0.302 | 7.413 | 1.930 | 4.807 | 0.401 | 22.000 |
| ICMA 843-22 | 56.670 | 39.630 | 0.248 | 7.340 | 0.467 | 4.783 | 0.098 | 21.667 |
| ICMA 94555 | 63.000 | 39.300 | 0.302 | 6.957 | 0.070 | 5.007 | 0.014 | 20.000 |
| HMS 7A | 36.667 | 42.467 | 0.229 | 7.573 | 0.537 | 3.610 | 0.148 | 10.000 |
| HMS 47A | 51.000 | 34.867 | 0.174 | 7.587 | 1.863 | 2.953 | 0.631 | 5.667 |
| ICMA 95222 | 71.333 | 34.267 | 0.315 | 6.353 | 1.840 | 4.010 | 0.459 | 11.000 |
| HBL 11 | 51.330 | 37.600 | 0.198 | 7.303 | 0.193 | 3.487 | 0.054 | 4.000 |
| H77/833-2-202 | 83.000 | 42.700 | 0.241 | 6.710 | 0.133 | 3.583 | 0.038 | 22.333 |
| AC 04-13 | 72.003 | 41.367 | 0.281 | 7.290 | 0.907 | 9.450 | 0.096 | 13.000 |
| HTP 94/54 | 81.330 | 38.163 | 0.186 | 5.877 | 0.163 | 5.047 | 0.033 | 4.000 |

*PH: plant height; SPAD: soil plant analysis development (SPAD) chlorophyll meter reading; gS: stomatal conductance; Pro: proline; Na^+^: sodium content; K^+^: potassium content; Na^+^/K^+^: sodium to potassium ratio; AGB: above ground biomass.*

**SUPPLEMENTARY TABLE 7c. Predicted grain yield and ranks of evaluated pearl millet hybrids at higher salinity level (EC_iw_ ~9 dSm^–1^) estimated through weighted coefficients (βs).**

| **Inbred lines** | **Constant**  **(α)** | **(-0.050)×**  **SPAD** | **0.050×**  **Pn** | **8.750×**  **gS** | **(-0.440)×**  **Pro** | **0.050×**  **TSP** | **(-0.180)×**  **TSS** | **0.380×**  **AGB** | **Predicted GY** | **Rank** |
| --- | --- | --- | --- | --- | --- | --- | --- | --- | --- | --- |
| HHB 67 Improved | -1.520 | -1.520 | 0.968 | 2.794 | -2.392 | 0.652 | -1.931 | 6.207 | 3.26 | 6 |
| HHB 146 |  | -1.952 | 0.770 | 3.620 | -3.155 | 0.512 | -2.002 | 10.767 | 7.04 | 1 |
| HHB 197 |  | -2.000 | 0.933 | 3.033 | -2.879 | 0.627 | -2.005 | 8.613 | 4.80 | 5 |
| HHB 226 |  | -1.578 | 0.646 | 3.141 | -2.788 | 0.577 | -2.775 | 6.967 | 2.67 | 7 |
| HHB 223 |  | -1.797 | 0.463 | 2.252 | -3.131 | 0.626 | -2.552 | 11.527 | 5.87 | 4 |
| HHB 234 |  | -2.158 | 0.388 | 2.774 | -3.137 | 0.603 | -2.219 | 12.287 | 7.02 | 2 |
| HHB 272 |  | -2.257 | 0.857 | 2.546 | -2.527 | 0.441 | -2.626 | 11.907 | 6.82 | 3 |
| Model Fitted: GY~ -1.520+ (-0.050)*PH + 0.050*SPAD+ 8.750*gS + (-0.440)*Pro + 0.050*SP + (-0.180)*SS+ 0.380*AGB | | | | | | | | | | |

*SPAD: soil plant analysis development (SPAD) chlorophyll meter reading; Pn: photosynthetic rate; gS: stomatal conductance; Pro: proline; TSP: total soluble protein; TSS: total soluble sugars; Na^+^: sodium content; K^+^: potassium content; Na^+^/K^+^: sodium to potassium ratio; AGB: above ground biomass; GY; grain yield.*

**SUPPLEMENTARY TABLE 7d.** **Predicted grain yield and ranks of evaluated pearl millet inbred lines at higher salinity level (EC_iw_ ~9 dSm^–1^) estimated through weighted coefficients (βs).**

| **Inbred lines** | **Constant (α)** | **0.014×**  **PH** | **0.031×**  **SPAD** | **3.418×**  **gS** | **0.156×**  **Pro** | **(-3.598)×**  **Na^+^** | **0.288×**  **K^+^** | **12.065×**  **Na^+^/K^+^** | **0.222×**  **AGB** | **Predicted GY** | **Rank** |
| --- | --- | --- | --- | --- | --- | --- | --- | --- | --- | --- | --- |
| ICMA 97111 | -6.560 | 1.596 | 1.432 | 1.033 | 1.156 | -6.944 | 1.384 | 4.838 | 4.884 | 2.82 | 4 |
| ICMA 843-22 |  | 0.793 | 1.229 | 0.849 | 1.145 | -1.679 | 1.378 | 1.186 | 4.810 | 3.15 | 3 |
| ICMA 94555 |  | 0.882 | 1.218 | 1.033 | 1.085 | -0.252 | 1.442 | 0.173 | 4.440 | 3.46 | 2 |
| HMS 7A |  | 0.513 | 1.316 | 0.783 | 1.181 | -1.931 | 1.040 | 1.790 | 2.220 | 0.35 | 6 |
| HMS 47A |  | 0.714 | 1.081 | 0.596 | 1.184 | -6.704 | 0.851 | 7.617 | 1.258 | 0.04 | 8 |
| ICMA 95222 |  | 0.999 | 1.062 | 1.077 | 0.991 | -6.620 | 1.155 | 5.538 | 2.442 | 0.08 | 7 |
| HBL 11 |  | 0.719 | 1.166 | 0.677 | 1.139 | -0.696 | 1.004 | 0.656 | 0.888 | -1.01 | 10 |
| H77/833-2-202 |  | 1.162 | 1.324 | 0.823 | 1.047 | -0.480 | 1.032 | 0.454 | 4.958 | 3.76 | 1 |
| AC 04-13 |  | 1.008 | 1.282 | 0.962 | 1.137 | -3.262 | 2.722 | 1.154 | 2.886 | 1.33 | 5 |
| HTP 94/54 |  | 1.139 | 1.183 | 0.636 | 0.917 | -0.588 | 1.453 | 0.394 | 0.888 | -0.54 | 9 |
| Model Fitted: GY~ -6.56 + 0.014*PH + 0.031*SPAD + 3.418*gS + 0.156*Pro + (-0.598)*Na^+^ + 0.288*K^+^ + 12.065*Na^+^/K^+^ + 0.222*AGB | | | | | | | | | | | |

*PH: plant height; SPAD: soil plant analysis development (SPAD) chlorophyll meter reading; gS: stomatal conductance; Pro: proline; Na^+^: sodium content; K^+^: potassium content; Na^+^/K^+^: sodium to potassium ratio; AGB: above ground biomass; GY; grain yield*
